# The Link Between Age and Partner Preferences in a Large, International Sample of Single Women

**DOI:** 10.1007/s12110-023-09460-4

**Published:** 2023-09-26

**Authors:** Laura J. Botzet, Amanda Shea, Virginia J. Vitzthum, Anna Druet, Maddie Sheesley, Tanja M. Gerlach

**Affiliations:** 1 Department for Biological Personality Psychology, University of Goettingen, Goettingen, Germany; 2 Leibniz Science Campus Primate Cognition, Deutsches Primatenzentrum GmbH, Goettingen, Germany; 3 Clue by BioWink GmbH, Berlin, Germany; 4 Evolutionary Anthropology Laboratory, Indiana University, Indianapolis, USA; 5 Department of Anthropology, Indiana University, Indianapolis, USA; 6 School of Psychology, Queen’s University Belfast, Belfast, UK

**Keywords:** Age, Ideal partner, Mate choice, Partner preferences, Parenting intention

## Abstract

Women’s capacity to reproduce varies over the life span, and developmental goals such as family formation are age-graded and shaped by social norms about the appropriate age for completing specific developmental tasks. Thus, a woman’s age may be linked to her ideas about what an ideal partner should be like. With the goals of replicating and extending prior research, in this study we examined the role of age in women’s partner preferences across the globe. We investigated associations of age with ideal long-term partner preferences in a cross-cultural sample of 17,254 single (i.e., unpartnered) heterosexual women, ages 18 to 67, from 147 countries. Data were collected via an online questionnaire, the Ideal Partner Survey. Confirming our preregistered hypotheses, we found no or only negligible age effects on preferences for kindness-supportiveness, attractiveness, financial security-successfulness, or education-intelligence. Age was, however, positively associated with preferences for confidence-assertiveness. Consistent with family formation goals, age was associated with an ideal partner’s parenting intentions (high until approximately age 30, then decreasing afterward). Age range deemed acceptable (and in particular, the discrepancy between one’s own age and the minimum ideal age of a partner) increased with age. This latter pattern also replicated in exploratory analyses based on subsamples of lesbian and bisexual women. In summary, age has a limited impact on partner preferences. Of the attributes investigated, only preference for confidence-assertiveness was linked with age. However, age range deemed acceptable and an ideal partner’s parenting intention, a dimension mostly neglected in earlier research, substantially vary with age.

**Supplementary Information:**

The online version contains supplementary material available at 10.1007/s12110-023-09460-4.

Beginning with Hill (1945), psychologists, and evolutionary psychologists in particular, have investigated adults’ preferences for an ideal partner, especially with regard to possible sex differences in such preferences (e.g., Buss, 1989; Buss et al., 1990, 2001; Feingold, 1992; Walter et al., 2020).^1^ In contrast, the possible effect of age on partner preferences has received much less attention.

There are several theoretical reasons for expecting age to influence partner preferences. First, mating motivations change with age. Whereas motivation for mate-seeking is significantly higher during emerging adulthood, motivations for mate retention and parenting become increasingly important as people age (Kenrick et al., 2010; Ko et al., 2020). Second, age-graded developmental goals, such as forming a family (Heckhausen et al., 2010), are also influenced by social norms and the fertility behavior of one’s social network (e.g., Keim et al., 2009). Third, from a biological perspective, age might be more important for female than for male humans because females’ ability to reproduce is not constant throughout adulthood. For female humans, the occurrence of menopause around the age of 50 (e.g., Bromberger et al., 1997; Palacios et al., 2010) constitutes a natural end to childbearing years and thus a real developmental deadline (Wrosch & Heckhausen, 2005). For male humans, even though there are also age-related changes in hormones (Harman et al., 2001), no such hard-and-fast developmental deadlines exist.

Empirically, evidence of age effects on partner preferences is mixed. Two studies conclude that there is no evidence for a substantial age effect on partner preferences (Schwarz & Hassebrauck, 2012; see also Alterovitz & Mendelsohn, 2011). Some researchers report a decrease in the preference for sex appeal (Menkin et al., 2015) and resource holding potential (Munro et al., 2014) as age increases.

According to Kenrick et al. (2010), the motivation for mate retention and especially parenting increases with age. Female humans are only fertile from menarche (typically occurring in their teens) to menopause (typically occurring around 50 years of age). Age at menarche has decreased over time in some populations (e.g., Wyshak & Frisch, 1982) and varies across populations (e.g., Udry & Cliquet, 1982). It ranges from 12 years of age in resource-rich populations such as the US (e.g., Biro et al., 2018) to 18 years of age in populations with fewer resources, such as rural Papua New Guinea (see Chester & Vitzthum, 2018, and Eveleth & Tanner, 1990, for more details on human reproductive maturation and growth). Female fertility peaks from about 20 to 30 years of age and ends with menopause (e.g., Bromberger et al., 1997; Palacios et al., 2010). Conception rates have been shown to decline after 30, and markedly so after age 35 (Balasch & Gratacós, 2012). From a biological perspective, it would therefore be reasonable for women to place a high importance on finding a partner who wants to be a parent during the years in which it is biologically possible for her to conceive and most likely to occur without requiring intervention. We therefore propose that the preference for a mate who wants to be a parent (i.e., parenting intention) follows the quadratic pattern observed for fertility. To our knowledge, and somewhat surprisingly, few previous studies have examined whether there are age-related differences in preference for parenting intention. In line with the theoretical considerations outlined above, Schaper (2019) found that age had a quadratic relationship with preference for a partner intending to parent in an age-diverse sample of 382 single, heterosexual participants: compared with 18- to 24-year-olds, the preference for intent to parent was higher for 25- to 34-year-olds and lower for 40- to 54-year-olds.

The ideal age of a potential partner also seems to vary with age. From an evolutionary perspective, male humans should prefer female humans in their peak reproductive years to maximize their own reproductive fitness (Kenrick & Keefe, 1992). Female humans desire older males in general (e.g., Schwarz & Hassebrauck, 2012) but outlive them in most cases (e.g., Luy & Gast, 2014). After a certain age, since fewer potential older partners are available for females, they must consider younger male humans as potential partners. In line with these theoretical considerations, Alterovitz and Mendelsohn (2011) showed that aging men desired women increasingly younger than themselves and women desired older men until age 75, after which they desired men younger than themselves. Similarly, Schwarz and Hassebrauck (2012) reported that with increasing age, women considered relatively younger men more acceptable and older men less acceptable. Conway et al. (2015) also reported that with increasing age, heterosexual men preferred younger partners; however, in their study, heterosexual women did not seem to express substantial interest in men younger than themselves.

Most of the literature investigating age effects on partner preferences has focused solely on heterosexual participants (e.g., Alterovitz, & Mendelsohn, 2011; Schwarz & Hassebrauck, 2012). To our knowledge, the only exception is the study by Conway et al. (2015), which differentiated between homosexual and heterosexual participants when investigating effects of own age on age preferences. Thus, it is largely unknown whether empirical patterns observed for nonheterosexual participants resemble patterns for heterosexual participants (either based on their own gender or the gender they are attracted to) and whether ideas focusing on heterosexual individuals based on evolutionary psychology generalize to those identifying as homosexual or bisexual.

## The Current Study

Based on a recently collected large international sample of single (i.e., unpartnered) women, the main goal of our study was to conceptually replicate and extend the study by Schwarz and Hassebrauck (2012). Those researchers investigated sex and age effects on partner preferences in a sample of 21,245 single, heterosexual participants from Germany (18–65 years old) using 82 mate selection criteria evaluated in earlier studies. Their findings were clear: sex was a major explanatory factor in partner preferences, but age explained very little of the variability in reported partner preferences.

Our study included partner preferences conceptually similar to those investigated by Schwarz and Hassebrauck (2012). In addition, however, we assessed the strength of respondents’ preference regarding parenting by asking them to rate (1) the importance of a partner sharing their preferred number of children and (2) the importance of a partner’s parenting intentions. Finally, we investigated age effects on the age range deemed acceptable (age of oldest and youngest partner deemed acceptable). All main analyses focused on heterosexual single women (note that participants were explicitly asked about their gender and not about their biological sex). We also explored the links between age and partner preferences, parenting intention, as well as age range in lesbian and bisexual single women.

We restricted our main analyses to heterosexual and single participants to replicate the study by Schwarz and Hassebrauck (2012) as closely as possible. We additionally restricted our analyses to women because of low participation rates of men. The age range of women was from 18 to around 50, the average age at menopause (e.g., Bromberger et al., 1997; Palacios et al., 2010). No data are available before the age of 18, meaning that we do not cover the very interesting phase of life from menarche (i.e., around 12 years of age in resource-rich populations; Biro et al., 2018), to the later phase of adolescence. In addition, very little data is available from women over the age of 50, therefore not adequately covering a second very interesting phase of life that includes developmental milestones such as becoming a grandparent, retirement, and the emergence of health issues. Nevertheless, this means the current study covers nearly the entire reproductive life span of women.

## Hypotheses

All hypotheses are summarized in Table 1. For verbatim hypotheses and specific operationalizations, see our preregistration at https://osf.io/qe3dr/.

**Table 1:** Hypotheses for partner preference attributes, parenting intention, and age range deemed acceptable

Hypothesis	Outcome		Age Effect	Expected Effect
H1a	Kindness-supportiveness	Importance	linear	none/negligible
H1b		Level	linear	none/negligible
H2a	Attractiveness	Importance	linear	none/negligible
H2b		Level	linear	none/negligible
H3a	Financially secure-successful	Importance	linear	none/negligible
H3b		Level	linear	none/negligible
H4a	Confidence-assertiveness	Importance	linear	none/negligible
H4b		Level	linear	none/negligible
H5a	Education-intelligence	Importance	linear	none/negligible
H5b		Level	linear	none/negligible
H6a	Importance of shared preference for number of children		linear	positive
H6b	Preferred level of partner’s intention to become a parent		linear	positive
H7a	Importance of shared preference for number of children		linear	positive
			quadratic	negative
H7b	Preferred level of partner’s intention to become a parent		linear	positive
			quadratic	negative
H8	Age range deemed acceptable		linear	none/negligible
H9	Youngest age deemed acceptable		linear	positive
H10	Oldest age deemed acceptable		linear	negative

Dovetailing with the results by Schwarz and Hassebrauck (2012), we expected no relationship between age and any of the psychological attributes summarized in H1–H5. Most studies about partner preferences have examined either importance ratings or preferred levels of an attribute. In our study, although we anticipated no differences between the two measurements, we included both scales and investigated them in parallel. Based on quadratic fertility patterns across the life span for women, we expected a positive linear relationship and a negative quadratic relationship between age and parenting intention (H6 and H7).^2^ Parenting intention was measured as (a) partner sharing the preference for number of children and (b) partner’s intention of becoming a parent. Following Schwarz and Hassebrauck (2012), we expected no relationship between age and age range deemed acceptable by women in an ideal partner (H8). We further expected that, with increasing age, women find younger men more acceptable (H9), while their acceptance for older men decreases (H10).

## Methods

This study is part of a larger project, the Ideal Partner Survey, for which supporting information can be found on the Open Science Framework (OSF): https://osf.io/wkzng/. The hypotheses and methods of the current study were preregistered at https://osf.io/qe3dr/. Deviations from our preregistration are noted in footnotes throughout the manuscript and described in more detail in the Electronic Supplemental Materials (ESM Table S1, inspired by Van’t Veer et al., 2019). The authors of the preregistration (i.e., first and last author of this manuscript) did not have access to the data prior to uploading the preregistration; the data-sharing agreement is available at https://osf.io/nvk5r/.

### Procedure

The study design was a cross-sectional online survey implemented in the survey software Typeform v2 (https://www.typeform.com/). The study was online from December 6 to December 21, 2018. Subjects were told that the survey was about the qualities of their ideal partner(s). Participants younger than 18 were not able to participate in the survey. All participants completed a 14-item section focusing on demographic information and long-term preferences. In a second step, they could answer an optional 30-item part (concerning past relationships and preferences for physical features). In a third part, participants could answer questions about potential short-term partners, if they were willing to do so. Ten parallel language versions of the survey were developed: Chinese, Danish, English, French, German, Italian,^3^ Japanese, Portuguese, Russian, and Spanish. Translators were native speakers of their respective languages who were contracted by the menstrual cycle tracking app Clue on a regular basis. A “four eyes” approach (one translator and one proofreader per language) was used. The survey was advertised via email campaigns to the respective user bases, messages within the Clue app, and social media channels.

### Variables

#### Exclusion Variables

Exclusion criteria as outlined below were based on the following variables. Gender was measured with a categorical item “*Do you identify as…*” with response options (a) *Woman*, (b) *Genderqueer/Nonbinary*, (c) *Man*, (d) *None of the above*, (e) *Prefer not to say*, and (f) *Other*. Sexual orientation was measured with a categorical item “*How would you describe your current sexual orientation?*” with response options (a) *Straight/Heterosexual*, (b) *Lesbian/Gay/Homosexual*, (c) *Bisexual/Pansexual*, (d) *Queer*, (e) *Asexual*, (f) *Prefer not to say*, and (g) *Other*. Relationship status was based on the item “*Select your relationships during the past 3 months*” with response options (a) *No romantic or sexual relationships during the past 3 months*, (b) *Short-term (casual) sexual relationship (e.g., hookups or one-night-stands)*, (c) *New (less than 1 month old) romantic and/or sexual relationship*, (d) *Ongoing (longer than 1 month) uncommitted/nonexclusive romantic and/or sexual relationship*, (e) *Long-term committed/exclusive sexual relationship with one or more partners*, and (f) *Other*. Multiple options could be chosen. Participants who chose response options (c), (d), (e), and/or (f) were treated as non-single. Seriousness was assessed with the categorical item “*We understand that sometimes people fill out questionnaires for fun and give answers that may not be accurate. In the interest of scientific accuracy, we will exclude those responses from our final analysis. Please choose one of the statements below*” with response options (a) *I took the survey seriously; please use my information in the study*, (b) *I did not answer seriously; please disregard my information*, and (c) *I choose not to answer*.

#### Variables Included in Analyses

Age was measured with one item asking “*How old are you?”* and participants answered with an integer number. Age was not restricted to a certain number; values higher than 100 were set to missing (affecting 15 participants in the raw dataset and 5 participants in the dataset after exclusion).^4^ All other variables and item wordings are listed in Table 2.

**Table 2:** Variables used for partner preference attributes, parenting intention, and age range

Attribute	Variable	Scale / Item
*For each trait below, first tell us how important it is to you when choosing an ideal long-term partner. Then tell us how much of the trait your ideal partner should have.*		
Kind	Importance	*Kind — How important is it to you?*
	Level	*Level of kindness — How kind should your partner be?*
Supportive	Importance	*Supportive — How important is it to you?*
	Level	*Supportive — How supportive should your partner be?*
Attractive body	Importance	*Attractive body — How important is it to you?*
	Level	*Attractive body — How attractive should your partner’s body be?*
Attractive face	Importance	*Attractive face — How important is it to you?*
	Level	*Attractive face — How attractive should your partner’s face be?*
Financially secure	Importance	*Financially secure — How important is it to you?*
	Level	*Financial security — How financially secure should your partner be?*
Successful/ambitious	Importance	*Successful/ambitious — How important is it to you?*
	Level	*Successful/ambitious — How successful/ambitious should your partner be?*
Confident	Importance	*Confident — How important is it to you?*
	Level	*Level of confidence — How confident should your partner be?*
Assertive	Importance	*Assertive — How important is it to you?*
	Level	*Level of assertiveness — How assertive should your partner be?*
Intelligence	Importance	*Intelligent — How important is it to you?*
	Level	*Level of intelligence — How intelligent should your partner be?*
Educated	Importance	*Educated — How important is it to you?*
	Level	*Level of education — How educated should your partner be?*
Parenting intention	Importance	*Shares my preference for number of children — How important is it to you?*
	Level	*Wants to be a parent — How much should your partner want to be a parent?*
Age range deemed acceptable	Minimum	*The acceptable age range for my long-term partner is at least* *__* *years old…*
	Maximum	*and no more than* *__* *years old.*

All importance items were measured on a scale from 0 *= not at all important* to 6 = *very important.* All level items were measured on a scale from (e.g.) 0 *= not kind* to 6 = *very kind*. Level of parenting intent was measured on a scale from 0 *= does not want* to 6 = *very much wants.* For each attribute, participants were only asked about the desired level if they had indicated an importance of at least 1. This means that participants who had indicated an importance of 0 were not asked about the desired level, implying a smaller sample size for those analyses. For the preference attributes, we calculated five dimensions using the mean of two items: *Kind-supportive* included the variables kind and supportive (*r*_importance_ = .24 [99.5% CI: .22, .26]; *r*_level_ = .28 [.26, .30]);^5^*attractiveness* included attractive face and attractive body (*r*_importance_ = .62 [.61, .64]; *r*_level_ = .59 [.57, .60]); *financially secure-successful* included financially secure and successful/ambitious (*r*_importance_ = .34 [.32, .36]; *r*_level_ = .35 [.33, .37]); *confident-assertive* included confident and assertive (*r*_importance_ = .29 [.27, .31]; *r*_level_ = .32 [.30, .34]); *intelligence-educated* included intelligence and educated (*r*_importance_ = .43 [.41, .45]; *r*_level_ = .46 [.44, .48]). We calculated all five dimensions separately for importance and for level ratings.

Age range deemed acceptable was assessed in the second part of the survey, which was optional. Therefore, sample size for this analysis might differ from those of other analyses. Age range deemed acceptable was calculated by subtracting minimum from maximum ideal age. Youngest age deemed acceptable was calculated by subtracting minimum ideal age from respondent’s age. Oldest age deemed acceptable was calculated by subtracting respondent’s age from maximum ideal age. Since minimum and maximum ideal age were restricted to a certain number, values higher than 100 were set to missing (affecting 2 and 1 participant[s], respectively, in the raw dataset and no participants in the dataset after exclusion).^6^ If minimum or maximum ideal age were missing, age range deemed acceptable was set as missing. If age range deemed acceptable was negative, values for age range deemed acceptable, youngest age deemed acceptable, and oldest age deemed acceptable were set as missing.

### Participants and Exclusion Criteria

**Fig. 1:**
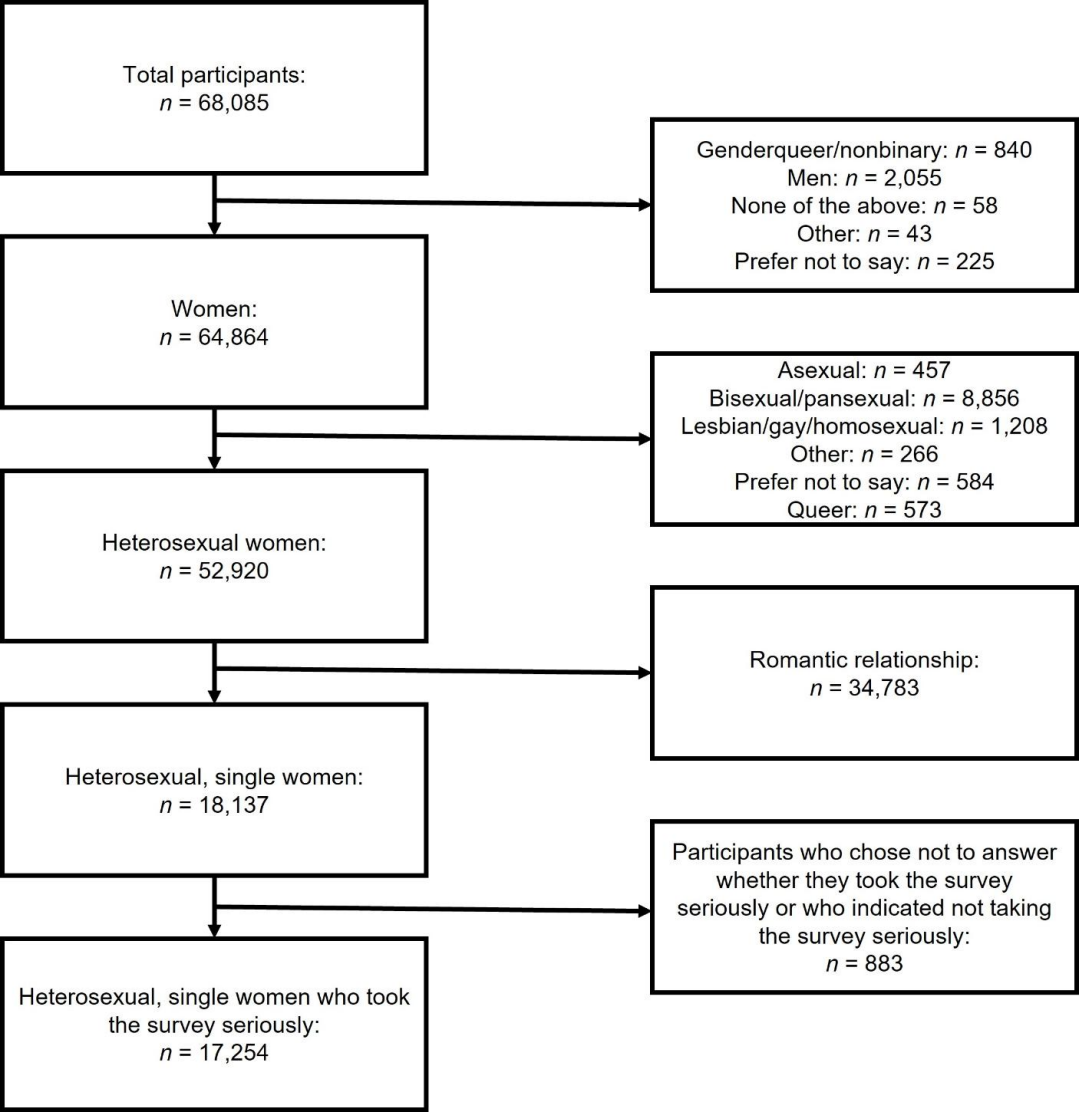
Exclusion steps for main analyses

68,085 people 18 years of age or older participated in the Ideal Partner Survey. Sample size was merely based on time constraints. For the current manuscript we excluded the following participants: (1) all participants who did not identify as women; (2) all participants who did not identify as heterosexual; (3) all participants who indicated being in a relationship or where it was not certain if participants were currently single; (4) all participants who did not answer the survey seriously or chose not to answer the seriousness question.^7^ We therefore excluded 50,831 participants (see Fig. 1), yielding a sample of 17,254 for the main analyses, with slightly differing sample sizes for each outcome. The exclusion process for our exploratory analyses is documented in the ESM and illustrated in Fig. S4.

Initially, we were interested in investigating age effects on partner preferences for women and men. We attempted to reach a sufficient number of male participants in addition to the predominantly female Clue user base by partnering with the condom company, myOne, for the distribution of our survey. However, before submitting our preregistration, it became evident that the sample of men would be insufficient for investigating potential small effects of age on partner preferences. Indeed, only 3% of the final sample were men (*n* = 2,055). Applying our exclusion criteria outlined above would have resulted in a sample size of 419 men from 27 countries. A large proportion of the men (83%) were from the United States, and their mean age was 32.49 (*SD* = 12.90; ranging from 18 to 85 years). We deemed this sample not suitable to test for age effects on partner preferences in a large, international sample paralleling our women’s sample and therefore decided to focus solely on women.

### Characteristics of the Current Sample

Overall, our sample consisted of 17,254 heterosexual single women with sample sizes ranging from 12,154 to 16,651 for specific analyses. Variation in the sample size between analyses can be explained by three reasons: (1) participants were included in the analyses even if they did not finish the survey, leading to missingness in the later parts of the survey; (2) participants were only asked about their desired level of a preference if they indicated an importance for this preference higher than 0, leading to missingness for the level items; and (3) random missingness due to participants not answering all questions shown.

**Fig. 2:**
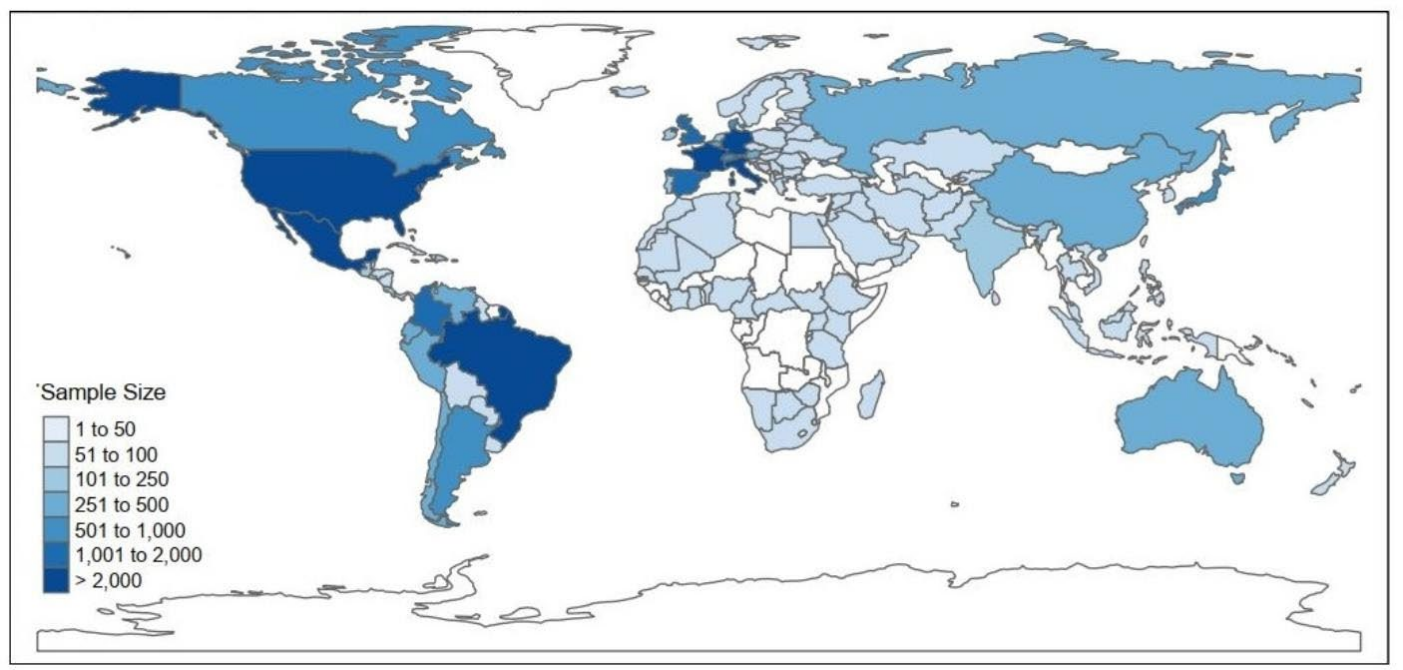
Country distribution for all women (*n* = 17,245). Unshaded countries indicate that no woman from this country was included in our analyses

Participants filled out the survey in 10 different languages as shown in Table 3. Fig. 2 shows the distribution of participants across 147 different countries (for more details see Table S2 in the ESM). On average, participants were 23.58 years old (SD *=* 6.92; ranging from 18 to 67 years). Table 4 displays means, standard deviations, and zero-order correlations for age and partner preferences including parenting intention and preference for age range deemed acceptable. Means and standard deviations as well as comparisons between groups based on sexual orientation can be found in Table S3 in the ESM.

**Table 3:** Language distribution for all women (*n* = 17,254)

Language	*n*	%	Language	*n*	%
Chinese	112	0.65	Italian	1,265	7.33
Danish	445	2.58	Japanese	349	2.02
English	3,656	21.19	Portuguese	1,098	6.36
French	3,224	18.69	Russian	253	1.47
German	2,601	15.07	Spanish	4,251	24.64

**Table 4:** Means, standard deviations, and zero-order correlation coefficients for age, partner preferences, and preference for ideal age range

	Variable	*M*	*SD*	(1)	(2)	(3)	(4)	(5)	(6)	(7)	(8)	(9)	(10)	(11)	(12)	(13)	(14)	(15)
(1)	Own Age	23.58	6.92															
(2)	Kindness-Supportiveness (I)	5.41	0.66	**.03**														
(3)	Kindness-Supportiveness (L)	5.15	0.70	**.04**	**.65**													
(4)	Attractiveness (I)	3.96	1.16	.01	**.05**	**.06**												
(5)	Attractiveness (L)	4.04	0.92	**−.03**	**.09**	**.13**	**.82**											
(6)	Financially Secure-Successful (I)	4.41	1.08	**.06**	**.15**	**.17**	**.27**	**.23**										
(7)	Financially Secure-Successful (L)	4.35	0.90	**.07**	**.15**	**.23**	**.23**	**.27**	**.80**									
(8)	Confidence-Assertiveness (I)	4.50	0.94	**.08**	**.18**	**.18**	**.12**	**.12**	**.31**	**.31**								
(9)	Confidence-Assertiveness (L)	4.40	0.85	**.11**	**.15**	**.24**	**.08**	**.13**	**.29**	**.36**	**.77**							
(10)	Education-Intelligence (I)	4.93	0.96	**.05**	**.12**	**.10**	**.21**	**.16**	**.40**	**.36**	**.25**	**.23**						
(11)	Education-Intelligence (L)	4.73	0.83	**.07**	**.11**	**.20**	**.17**	**.22**	**.36**	**.42**	**.28**	**.32**	**.75**					
(12)	Importance of shared preference for number of children	3.47	1.96	**.05**	**.11**	**.09**	**.09**	**.07**	**.18**	**.13**	**.05**	**.05**	**.08**	**.07**				
(13)	Preferred level of partner’s intention to become a parent	4.00	1.82	**−.10**	**.13**	**.14**	**.06**	**.07**	**.10**	**.10**	**.02**	**.03**	−.02	−.001	**.14**			
(14)	Ideal age range	8.12	6.23	**.33**	**−.03**	**−.06**	**−.03**	**−.06**	**−.06**	**−.06**	**−.09**	**−.08**	**−.04**	**−.05**	−.001	**−.08**		
(15)	Youngest age deemed acceptable relative to own age	-1.21	5.37	**−.39**	**.02**	**.02**	.01	.01	**.07**	**.06**	**.09**	**.08**	**.02**	**.02**	**−.03**	**.05**	**−.19**	
(16)	Oldest age deemed acceptable relative to own age	6.91	7.43	−.01	−.01	**−.03**	**−.02**	**−.04**	.003	−.01	−.01	−.01	−.02	**−.03**	**−.02**	**−.03**	**.70**	**−.57**

*Note.***Bold** correlation coefficients differ significantly from 0 (*p* < .05)

I = Importance rating of partner preference, L = Preferred level of partner preference

### Analyses

#### Statistical Models

Hypotheses were tested via multilevel regression modeling, using the statistical software R 4.2.0 (R Core Team, 2013) and the packages *dplyr* 1.0.9 (Wickham et al., 2022), *ggplot2* 3.3.6 (Wickham, 2016), *effects* 4.2-1 (Fox & Weisberg, 2019), *effectsize* 0.7.0 (Ben-Shachar et al., 2020), *lmerTest* 3.1-3 (Kuznetsova et al., 2017), *lme4* 1.1–29 (Bates et al., 2015), and *sjstats* 0.18.1 (Lüdecke, 2021). All analyses included age as a predictor (and a quadratic age effect for hypotheses H7a and H7b) and a random intercept for country.^8^ To standardize beta coefficients, we used the function *standardize_parameters* from the package *effectsize* with the method *basic* (Ben-Shachar et al., 2020).

#### Inference Criteria

The main inference criterion was the *p*-value. Echoing calls for more stringent significance cutoffs and in light of the expected large sample size, we set the significance threshold to .005 (Benjamin et al., 2018). In addition, we compared effect sizes with those previously reported in the literature. In Schwarz and Hassebrauck (2012), effect sizes (standardized beta coefficients) for age on partner preferences ranged from β *=* −0.001 to 0.159 (in a model with sex, age, and their interaction as predictors). Based on their analyses, we concluded that effect sizes smaller than |β*| =* 0.10 were negligible. A β of ± 0.10 corresponds to a change of ± 0.10 standard deviation in the outcome variable when the predictor variable changes by 1 standard deviation. Table 5 summarizes our inference criteria for linear age effects.

**Table 5:** Inference criteria for linear age effects

	None/Negligible	Undecidable	Substantial
*p*-value	—	—	< .005
absolute value of β	< 0.10 CI excluding ± 0.10	CI including ± 0.10	> 0.10 CI excluding ± 0.10

*Note:* β = standardized beta coefficient, CI = 99.5% confidence interval

#### Robustness and Exploratory Analysis

In preregistered robustness checks, we performed analyses with a random intercept for language instead of country to account for potential differences in translation. In a second robustness check that was not preregistered, we performed all analyses excluding women with missing answers to the seriousness question (*n* = 1,952). To include all possible combinations, we performed a third robustness check combining the first and second (random intercept for language and excluding women with missing answers to the seriousness question).

We investigated the links between age and partner preferences including parenting intention and age range deemed acceptable in an exploratory manner based on the subsamples of lesbian (*n* = 467) as well as bisexual (*n* = 3,085) women following the same plan as for the analyses outlined above. All exploratory results can be found in the ESM (Tables S4–S9 and Figs. S5–S14).

### Availability of Data, Code, and Analyses

Since we cannot share the data publicly, we uploaded a synthetic dataset created with the R-package *synthpop* (Nowok et al., 2016) based on the sample included in our analyses to the OSF (https://osf.io/qkr9a/). The synthetic dataset mimics many of the central features of the real data, including means and bivariate associations, and thus can be used by others to check our code and to test their own hypothesis. The analysis code is uploaded as part of the accompanying project on the OSF (https://osf.io/mxf9p/). In addition, a codebook generated with the R-package *codebook* (Arslan, 2019) is available.

## Results

### Age Effects on Partner Preference Attributes

**Fig. 3:**
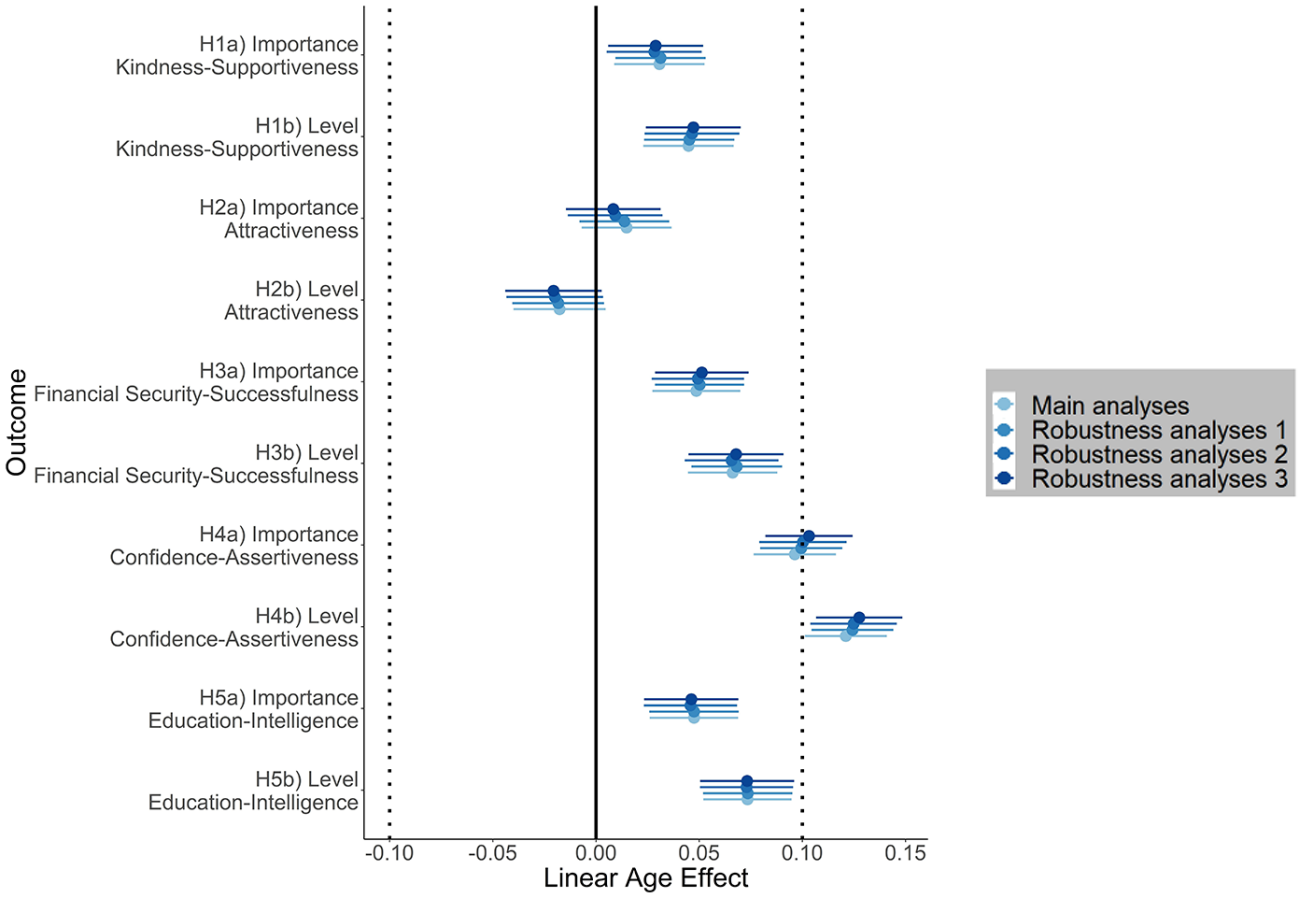
Effect size estimates of linear age effects on partner preference attributes for main analyses and three robustness analyses. Partner preferences include preferences for kindness-supportiveness (H1a, H1b), attractiveness (H2a, H2b), financial security-successfulness (H3a, H3b), confidence-assertiveness (H4a, H4b), and education-intelligence (H5a, H5b). Robustness analyses 1: random intercept for language instead of countries; robustness analyses 2: excluding women with missing answers to the seriousness question (*n*_excluded_ = 1,952); robustness analyses 3: random intercept for language instead of countries and excluding women with missing answers to the seriousness question (*n*_excluded_ = 1,952). Graphs display standardized linear age effects, error bars represent 99.5% confidence intervals. Dotted lines indicate the inference criteria for substantial effects *(p* < .005 and |β| > 0.10 with CIs excluding ± 0.10*)*

Table 6 summarizes the main results for age effects on partner preferences. Effect size estimates for main analyses and robustness analyses are displayed in Fig. 3. Applying our inference criteria outlined in Table 3, we found no or only negligible age effects on kindness-supportiveness (H1a (importance): β = 0.03 [99.5 CI: 0.01, 0.05], H1b (level): β = 0.04 [0.02, 0.07]), attractiveness (H2a (importance): β = 0.01 [− 0.01, 0.04], H2b (level): β = −0.02 [− 0.04, 0.005]), financial security-successfulness (H3a (importance): β = 0.05 [0.03, 0.07], H3b (level): β = 0.07 [0.04, 0.09]), and education-intelligence (H5a (importance): β = 0.05 [0.03, 0.07], H5b (level): β = 0.07 [0.05, 0.09]). Based on our inference criteria, we could not determine whether a positive linear age effect on importance of confidence-assertiveness exists (H4a: β = 0.10 [0.08, 0.12]), but there was evidence for a positive linear age effect on level of confidence-assertiveness (H4b: β = 0.12 [0.10, 0.14]). Fig. S1 in the ESM illustrates linear age effects on partner preferences. In summary, we found that older women preferred a higher level of confidence-assertiveness (and presumably also placed a higher importance on confidence-assertiveness) in their ideal partner than younger women, but we found no support for other substantial effects of age on partner preference attributes.

**Table 6:** Main results for age effects on partner preferences including preferences for kindness-supportiveness, attractiveness, financial security-successfulness, confidence-assertiveness, and education-intelligence

Hypothesis	Outcome		*n*	Countries	Linear Age Effect *b* [CI]	Linear Age Effect β [CI]	*p*
H1a	Kindness-supportiveness	Importance	16,185	146	0.003 [0.001, 0.01]	0.03 [0.01, 0.05]	< .001
H1b		Level	16,072	146	0.005 [0.002, 0.01]	0.04 [0.02, 0.07]	< .001
H2a	Attractiveness	Importance	16,399	147	0.002 [− 0.001, 0.01]	0.01 [− 0.01, 0.04]	.06
H2b		Level	15,761	146	−0.002 [− 0.01, 0.001]	−0.02 [− 0.04, 0.005]	.03
H3a	Financially secure-successful	Importance	16,368	147	0.01 [0.004, 0.01]	0.05 [0.03, 0.07]	< .001
H3b		Level	15,762	146	0.01 [0.004, 0.01]	0.07 [0.04, 0.09]	< .001
H4a	Confidence-assertiveness	Importance	16,508	147	0.01 [0.01, 0.02]	0.10 [0.08, 0.12]	< .001
H4b		Level	16,132	147	**0.01** **[0.01, 0.02]**	**0.12** **[0.10, 0.14]**	< .001
H5a	Education-intelligence	Importance	16,473	147	0.01 [0.004, 0.01]	0.05 [0.03, 0.07]	< .001
H5b		Level	16,104	142	0.01 [0.01, 0.01]	0.07 [0.05, 0.09]	< .001

*Note*. All analyses included a linear effect of age as a predictor and a random intercept for country. Sample size and number of countries differ for reasons explained in detail in the text. For all hypotheses the raw (*b*) and standardized (β) beta coefficients for the linear effect of age are displayed. **Bold** effect size estimates indicate substantial effects (*p* < .005 and |β| > 0.10 with CIs excluding ± 0.10)

### Age Effects on Parenting Intention

**Fig. 4:**
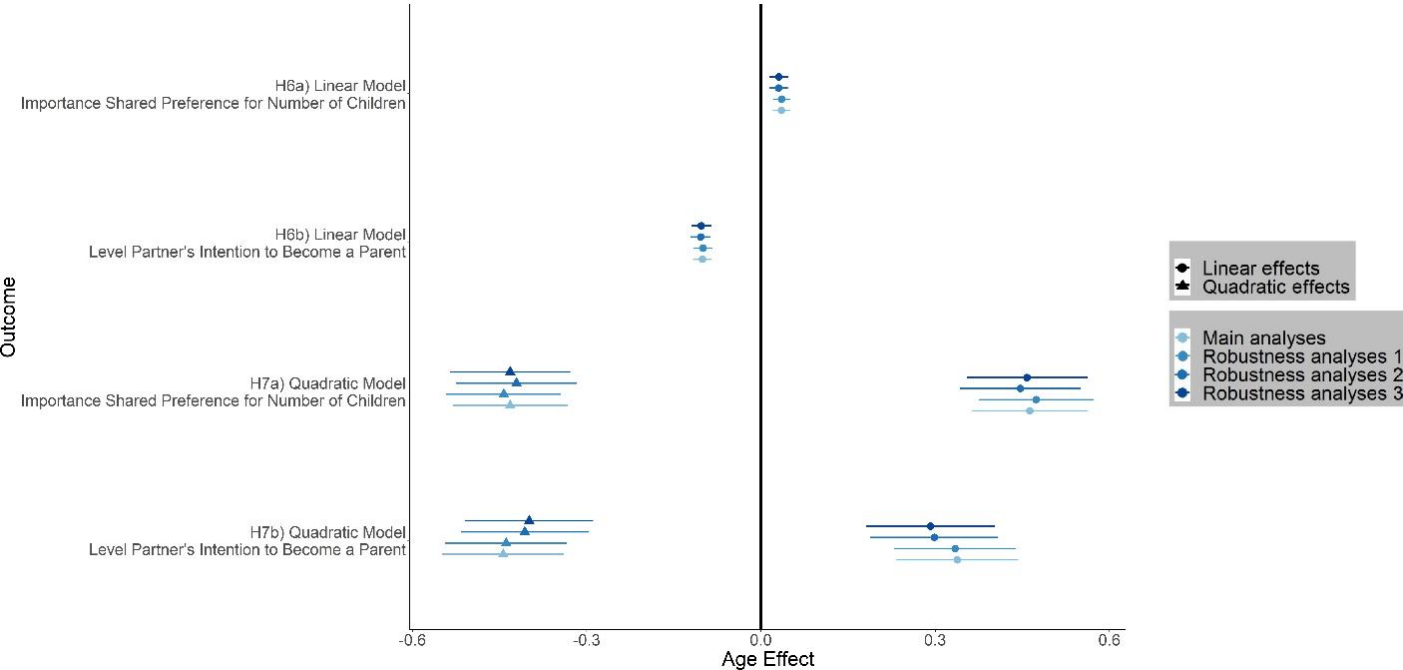
Effect size estimates of linear and quadratic age effects on preference for parenting intention for main analyses and three robustness analyses. Robustness analyses 1: random intercept for language instead of countries; robustness analyses 2: excluding women with missing answers to the seriousness question (*n*_excluded_ = 1,952); robustness analyses 3: random intercept for language instead of countries and excluding women with missing answers to the seriousness question (*n*_excluded_ = 1,952). Graphs display standardized linear and quadratic age effects, error bars represent 99.5% confidence intervals. Confidence intervals for effects estimates based on linear models are narrower compared to confidence intervals for effect estimates based on quadratic models because including a quadratic term decreases accuracy of estimates. In the models including only the linear predictor the effect of age never reached substantiality (*p* < .005 and |β| > 0.10 with CIs excluding ± 0.10)

**Fig. 5:**
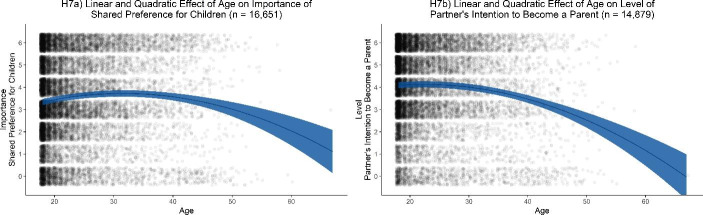
Preference for parenting intention by a linear and quadratic effect of age controlled for a random intercept for country. Parenting intention includes importance rating for a partner sharing the preference for number of children (H7a) and preference for the level of partner’s intention to become a parent (H7b). Blue areas represent 99.5% confidence intervals

Table 7 summarizes our main results for age effects on parenting intention. Fig. 4 shows linear and quadratic effect size estimates of age on parenting intention for main analyses and robustness analyses. We found no evidence for a substantial linear age effect on importance of a shared preference for number of children (H6a: β = 0.04 [0.02, 0.06]). Considering our inference criteria, we could not determine whether the negative linear age effect on preferred level of partner’s intention to become a parent was substantial or negligible (H6b: β = −0.10 [− 0.12, − 0.08]). In the model including a linear and a quadratic age effect on importance of a shared preference for number of children, we found evidence for a positive linear age effect (H7a: β = 0.46 [0.32, 0.60]) and a negative quadratic age effect (H7a: β = −0.43 [− 0.57, − 0.29]). These results are consistent with the model including a linear and a quadratic age effect on preferred level of partner’s intention: We found evidence for a positive linear age effect (H7b: β = 0.34 [0.19, 0.49]) and a negative quadratic age effect (H7b: β = −0.44 [− 0.59, − 0.29]). Fig. 5 illustrates linear and quadratic age effects on parenting intention. In summary, our results point to an inverted U-shaped pattern between age and preference for parenting intention: Both preference for partner’s level of intent to become a parent and importance of shared preference for number of children increased until a certain age, yet afterwards declined. However, our preregistered analyses provided no information about the exact tipping points.

**Table 7:** Main results for age effects on parenting intention

Hypothesis	Outcome	*n*	Countries	Predictor	Age Effect *b* [CI]	Age Effect β [CI]	*p*
H6a	Importance of shared preference for number of children	16,651	147	linear	0.01 [0.004, 0.02]	0.04 [0.01, 0.06]	< .001
H6b	Preferred level of partner’s intention to become a parent	14,879	140	linear	−0.03 [− 0.03, − 0.02]	−0.10 [− 0.12, − 0.08]	< .001
H7a	Importance of shared preference for number of children	16,651	147	linear	0.13 [0.09, 0.17]	0.46 [0.32, 0.60]	< .001
				quadratic	−0.002 [− 0.003, − 0.001]	−0.43 [− 0.57, − 0.29]	< .001
H7b	Preferred level of partner’s intention to become a parent	14,879	140	linear	0.09 [− 0.05, − 0.13]	0.34 [0.19, 0.49]	< .001
				quadratic	−0.002 [− 0.003, − 0.001]	−0.44 [− 0.59, − 0.29]	< .001

*Note*. Models for hypotheses H6a and H6b included age as a linear predictor and a random intercept for country. Models for hypotheses H7a and H7b included age as a linear and as a quadratic predictor and a random intercept for country. Sample size and number of countries differ for reasons explained in detail in the text. For all hypotheses except for H7a and H7b the raw (*b*) and standardized (β) beta coefficients for the linear effect of age are displayed. For H7a and H7b the raw and standardized beta coefficients for the quadratic effect of age are displayed. In the models including only the linear predictor, the effect of age never reached substantiality (*p* < .005 and |β| > 0.10 with CIs excluding ± 0.10)

During data analysis—but after writing the preregistration—we became aware of the two-lines approach by Simonsohn (2018). Simonsohn (2018) argues that applying quadratic regressions for testing U-shaped patterns—as we did for our initial analyses—can lead to a 100% false-positive rate, and he introduced the two-lines approach as an alternative for testing U-shaped relationships between variables. In the current study, we used this approach to more exactly pinpoint the age at which the relationship between age and parenting intention reverses. The two-lines approach estimates two regression lines (one for low and one for high values of the predictor). The breakpoint is determined by applying the Robin Hood algorithm. We performed the two-lines approach using R code provided at http://webstimate.org/twolines/. We predicted the importance of parenting intention and the preferred level of parenting intention by age. Of note, it is not possible to use the current two-lines approach with multilevel models; therefore, the results are controlled for neither a random intercept nor a random slope for country. In addition, we report unstandardized beta coefficients. The importance of parenting intention increased with age until age 30 (*b* = 0.04, *t*_16,647_ = 8.50, *p* < .001) and decreased afterwards (*b* = − 0.03, *t*_16,647_ = − 4.99, *p* < .001). The preferred level of parenting intention stayed consistent with increasing age until age 28 (*b* = − 0.01, *t*_14,875_ = − 1.61, *p* = .24) and decreased afterwards (*b* = − 0.06, *t*_14,875_ = − 11.14, *p* < .001). Fig. S2 (importance of parenting intention) and Fig. S3 (preferred level of parenting intention) in the ESM display the analyses graphically. To summarize, in the current study, women’s preferences for a partner’s parenting intention increased until approximately age 30, but then declined.

### Age Effects on Age Range Deemed Acceptable

**Fig. 6:**
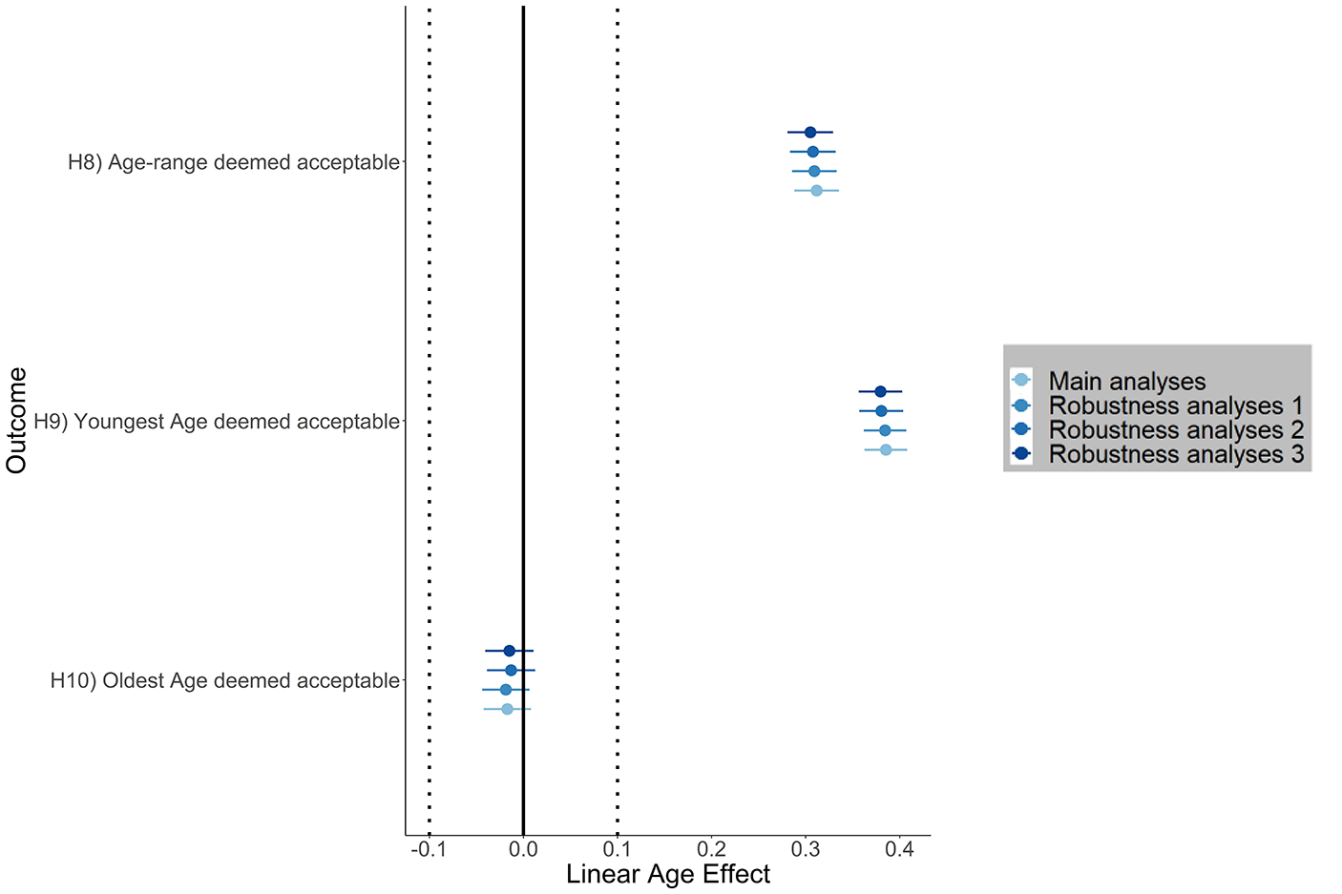
Effect size estimates of linear effects on age range deemed acceptable and on youngest and oldest age deemed acceptable for main analyses and three robustness analyses. Robustness analyses 1: random intercept for language instead of countries; robustness analyses 2: excluding women with missing answers to the seriousness question (*n*_excluded_ = 1,952); robustness analyses 3: random intercept for language instead of countries and excluding women with missing answers to the seriousness question (*n*_excluded_ = 1,952). Graphs display standardized linear effects, error bars represent 99.5% confidence intervals. Dotted lines indicate the inference criteria for substantial effects *(p* < .005 and |β| > 0.10 with CIs excluding ± 0.10*)*

**Fig. 7:**
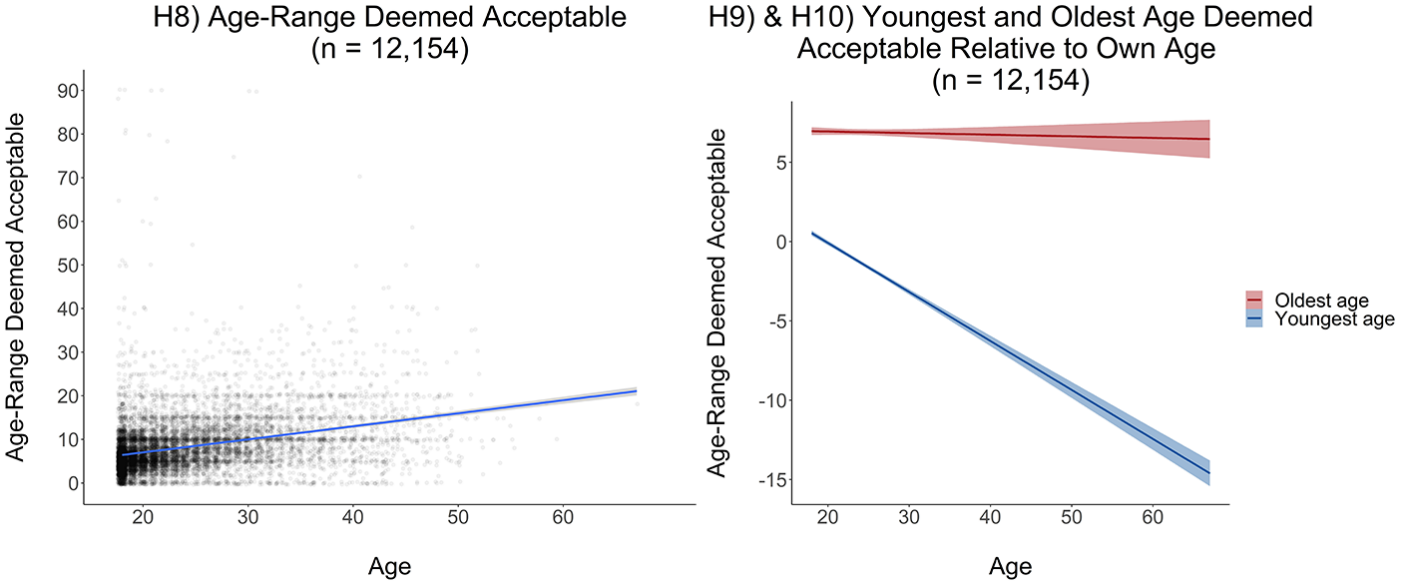
Age range deemed acceptable and youngest and oldest age deemed acceptable by own age. Graphs display means and 99.5% confidence intervals

Table 8 summarizes our main results for age effects on ideal age range. Fig. 6 shows linear effect size estimates of age on age range deemed acceptable and on youngest and oldest age deemed acceptable. We found a positive linear age effect on age range deemed acceptable (H8: β = 0.31 [0.29, 0.34]), a positive effect on youngest age deemed acceptable (H9: β = 0.39 [0.36, 0.41]), but no evidence for an age effect on oldest age deemed acceptable (H10: β = −0.02 [− 0.04, 0.01]). Fig. 7 illustrates linear age effects on age range deemed acceptable and youngest and oldest age deemed acceptable. We found that older women indicated a broader age range deemed acceptable in their ideal partner than younger women. This increase in the age range deemed acceptable could be explained by a higher flexibility in older women concerning the youngest age deemed acceptable relative to their own age.

**Table 8:** Main results for age effects on ideal age range

Hypothesis	Outcome	*n*	Countries	Linear Age Effect *b* [CI]	Linear Age Effect β [CI]	*p*
H8	Age range deemed acceptable	12,154	139	**0.28** **[0.26, 0.31]**	**0.31** **[0.29, 0.34]**	< .001
H9	Youngest age deemed acceptable	12,154	139	**0.30** **[0.29, 0.32]**	**0.39** **[0.36, 0.41]**	< .001
H10	Oldest age deemed acceptable	12,154	139	−0.02 [− 0.05, 0.01]	−0.02 [− 0.04, 0.01]	.06

*Note*. All analyses included a linear effect of age as a predictor and a random intercept for country. Sample size and number of countries differ for reasons explained in detail in the text. For all hypotheses the raw (*b*) and the standardized (β) beta coefficients for the linear effect of age are displayed. **Bold** effect size estimates indicate substantial effects (*p* < .005 and |β| > 0.10 with CIs excluding ± 0.10)

### Age Effects in Lesbian and Bisexual Women

The following results for lesbian and bisexual women are based on exploratory analyses (i.e., we did not specify any hypotheses). They should therefore be seen as preliminary and be used to inform future research focusing on nonheterosexual individuals. Descriptive information as well as comparisons between groups based on sexual orientation can be found in Table S3. In addition, all results for our exploratory analyses can be found in detail in the ESM (Tables S4–S9 and Figs. S5–S14).

The subsample of women identifying as lesbian likely was too small (*n* ranging from 331 to 456) to determine whether age played a substantial role in shaping preference attributes (Table S4 and Fig. S5). No clear pattern was apparent for the link between age and parenting intention (Table S5, Figs. S6 and S7), yet age range deemed acceptable increased with age (Table S6, Figs. S8 and S9). As in the heterosexual sample, this was due to older women lowering the youngest age deemed acceptable in an ideal partner.

In women identifying as bisexual (*n* ranging from 2,239 to 3009), age was not linked to desired level of attractiveness, nor was it linked to importance and desired level of financial security and successfulness. For all other preference attributes, the subsample of bisexual women was too small to reach a definitive conclusion (Table S7 and Fig. S10). While the pattern for the relationship between age and parenting intention was mixed for bisexual women (Table S8, Figs. S11 and S12), for age range, the same pattern as for heterosexual and lesbian women was apparent: older women increased their range by being more accepting toward younger potential partners (Table S9, Figs. S13 and S14).

## Discussion

The main goal of this study was to conceptually replicate and extend the study by Schwarz and Hassebrauck (2012) investigating age effects on partner preferences. Analyses were based on a large international sample of heterosexual single women, and extensive robustness checks were provided. Decisions on hypotheses for age effects on partner preference attributes, parenting intention, and age range deemed acceptable are summarized in Table 9. We found no evidence for substantial age effects on any of the partner preference attributes except for a positive association between age and confidence-assertiveness. Considering the relationship between age and parenting intention, analyses suggested a linear and a quadratic relationship. In addition, higher age was linked to a broader age range deemed acceptable, with an increased acceptance for younger partners but constant levels of acceptance for older partners. This latter pattern was also apparent in exploratory analyses of lesbian and bisexual women.

**Table 9:** Decision on hypotheses for age effects on partner preference attributes, parenting intention, and age range deemed acceptable

Hypothesis	Outcome		Age Effect	Expected Effect	Empirical Effect	Hypothesis Confirmed?
H1a	Kindness-supportiveness	Importance	linear	none/negligible	none/negligible	✓
H1b		Level	linear	none/negligible	none/negligible	✓
H2a	Attractiveness	Importance	linear	none/negligible	none/negligible	✓
H2b		Level	linear	none/negligible	none/negligible	✓
H3a	Financially secure-successful	Importance	linear	none/negligible	none/negligible	✓
H3b		Level	linear	none/negligible	none/negligible	✓
H4a	Confidence-assertiveness	Importance	linear	none/negligible	potentially positive	undecidable
H4b		Level	linear	none/negligible	positive	X
H5a	Education-intelligence	Importance	linear	none/negligible	none/negligible	✓
H5b		Level	linear	none/negligible	none/negligible	✓
H6a	Importance of shared preference for number of children		linear	positive	none/negligible	X
H6b	Preferred level of partner’s intention to become a parent		linear	positive	potentially negative	X
H7a	Importance of shared preference for number of children		linear	positive	positive	✓
			quadratic	negative	negative	✓
H7b	Preferred level of partner’s intention to become a parent		linear	positive	positive	✓
			quadratic	negative	negative	✓
H8	Age range deemed acceptable		linear	none/negligible	positive	X
H9	Youngest age deemed acceptable		linear	positive	positive	✓
H10	Oldest age deemed acceptable		linear	negative	none/negligible	X

✓ = hypothesis confirmed; X = hypothesis rejected

### Partner Preference Attributes

Supporting our hypotheses H1, H2, H3, and H5, we found evidence for no or only negligible relationships between age and almost all preference attributes, measured as both importance ratings and preferred levels of attributes. Contrary to our hypotheses H4a and H4b, we found support for a positive relationship between age and confidence and assertiveness.

These results conceptually replicate previous findings by Schwarz and Hassebrauck (2012) with one exception. Although we found a positive relationship between age and the preference for confidence-assertiveness (potentially positive for importance of confidence-assertiveness and clearly positive for the level of confidence-assertiveness), the original study found no relationship between age and conceptually similar constructs such as being dominant, cultivated, or humorous. According to Abele (2003), confidence and assertiveness are stereotypically male characteristics. One possibility is that women’s preferences for these characteristics indeed increase with age. However, given the cross-sectional nature of our data, a cohort effect with the youngest women in the sample simply being less attracted to such stereotypically male characteristics seems equally plausible. Future research utilizing longitudinal data is needed to separate age effects from cohort effects.

In our study, to replicate the work by Schwarz and Hassebrauck (2012) as closely as possible, we focused on single women only. However, excluding women who currently are in a relationship might have influenced our results. For instance, a longitudinal study by Gerlach et al. (2019) found that participants who entered a relationship adjusted their partner preferences to the characteristics of their partner. Importantly, the same study also showed that participants who were still single after several months lowered their expectations regarding an ideal partner, compared with those who had entered a relationship in the meanwhile. It can further be speculated that relationship status could make a larger difference for older as opposed to very young women, because relationships for women in their early twenties might be more fleeting, thus exerting less of an influence on preferences. With increasing age, relationships tend to be more stable and committed, thereby likely exerting more of an influence on those within relationships, but perhaps also prompting women who have not yet secured a partner to lower their expectations. If this is indeed the case, results based on our subsample of single women might underestimate potential effects of age on partner preferences in women from the general population. While it is important to note that our results should not be generalized beyond single women, we invite future studies to obtain more information about participants’ relationship histories and romantic success—for example, by including event history calendars (Driebe et al., 2023; Wieczorek et al., 2020).

### Parenting Intention

Although we found no support for a positive linear age effect on parenting intention—measured as (a) partner sharing the preference for number of children and (b) partner’s intention of becoming a parent—in models including only a linear age effect (H6a and H6b), our hypotheses expecting a positive linear relationship and a negative quadratic relationship between age and parenting intention were supported in the models including a linear and a quadratic age effect (H7a and H7b).

An evolutionary perspective would likely have predicted a decrease in preference for parenting intention to start around the onset of menopause (after 50) or the years immediately preceding it. Research on women’s “biological clock” contends that, psychologically, age 40 likely is the age that is perceived as the developmental deadline for childbearing (see Heckhausen et al., 2001). While modeling the relationship of age and preference for parenting intention with quadratic effects left us unable to determine an exact tipping point, interestingly, applying the two-lines approach by Simonsohn (2018) showed that the decrease already begins around age 28 to 30. One explanation could be that the overwhelming majority of young women start off with the notion that they will want to start a family at some point in the future. Yet, when life progresses and life plans become more concrete, a notable share of women may reconsider, either postponing parenthood to prioritize other endeavors (Spéder & Kapitány, 2009) or even concluding that a life without children is an attractive option (Stahnke et al., 2022). Alternatively, older women are simply more likely to already have children from earlier relationships and thus may ascribe less importance to a partner’s parenting intention or even prefer a partner with lower parenting intentions. Unfortunately, since participants were not asked in the survey whether they already had children, we could not disentangle these possibilities.

### Age Range

Contrary to our hypothesis H8, we found a positive relationship between age and age range deemed acceptable. This was due to the fact that we found a positive relationship between age and youngest age deemed acceptable (H9), but none or only a negligible relationship between age and oldest age deemed acceptable (H10). While women accepted younger men with increasing age, their acceptance for older men stayed consistent. The same pattern was also observed in lesbian and bisexual women (see ESM).

The original study by Schwarz and Hassebrauck (2012) reported an increase in acceptance for younger men with age, while female acceptance for older men decreased. We replicated the increase in youngest age deemed acceptable yet found no evidence for a decrease in oldest age deemed acceptable. When interpreting these results, one needs to bear in mind the age distribution in our sample. The age range in the current study (18‒67 years) is comparable to the age range from 18 to 65 years in the study by Schwarz and Hassebrauck (2012). However, mean age differed by 17 years (*M* = 24 and 41 years, respectively), and whereas age was normally distributed in Schwarz and Hassebrauck (2012), the distribution in the current sample was right (i.e., positively) skewed (median = 21, mode = 20, skewness ɣ = 1.59). Therefore, the share of older women might not have been sufficient to uncover potential age effects in this subsample. In sum, and contrasting with Schwarz and Hassebrauck’s (2012) sample, the current sample may not have included enough women for whom concerns regarding an older partner’s health and its possible impact on a couple’s everyday life (including leisure activities) might already have been salient.

### Evidence of Absence or Absence of Evidence?

Different theoretical perspectives lead to hypothesizing potential age effects on partner preferences. Taking together empirical work by Schwarz and Hassebrauck (2012) and the current study, evidence for the presence of age effects is very limited and tied to specific attributes, such as confidence-assertiveness. However, analyses suggested that age is important in shaping women’s preferences for parenting intention, with a tipping point at around 30 years of age. This pattern could be explained by a corrosion of the default (positive) parenting intention preference in younger women and a potential decrease in preference for partner’s parenting intention after having children. Further, with increasing age, women seem to become more flexible regarding the age range deemed acceptable in an ideal partner. This could be explained by a smaller mating market in older cohorts (many same-aged or older men after the age of 30 are already in committed, romantic relationships) and the perceived need to widen the range of potential acceptable partners (see Sassler, 2010).

Nevertheless, by setting predefined interference criteria to judge whether an effect can be considered substantial, the current study provides evidence for an absence of age effects on many partner preferences. Yet, while our study adds to the growing body of research showing that age plays no or only a negligible role in shaping many partner preferences, preferences for confidence-assertiveness, parenting intention, and age range deemed acceptable in an ideal partner were notable exceptions.

### Similarities and Differences Based on Sexual Orientation

Since little is known about the effect of age on partner preferences in nonheterosexual women, our analyses investigating these effects in lesbian and bisexual women were exploratory. Our results for lesbian and bisexual women confirmed that large sample sizes are needed to determine whether potentially small effects of age on partner preferences are substantial or negligible. Overall, just as in heterosexual women, there was no empirical evidence that age plays an important role in shaping preferences for specific attributes. We did not find the substantial positive effect of age on the preference for confidence-assertiveness in lesbian or bisexual women. Stereotypically male characteristics such as confidence and assertiveness appear to be overall less important for nonheterosexual women in general (see Table S3), and their importance does not increase with age for lesbian or bisexual women.

We found no evidence for linear or quadratic effects of age on preferences for parenting intention in lesbian individuals, potentially indicating that reproductive goals are less salient for these women (see Table S3). For bisexual women, the pattern for age effects on preference for parenting intention resembled the pattern observed in heterosexual women, even though only the linear age effects were significant.

For all three groups, the age range deemed acceptable increased substantially with age because women accepted younger partners with increasing age while their acceptance for older partners stayed relatively consistent. Overall, the age range deemed acceptable appeared to be larger for lesbian and bisexual women across all ages compared with heterosexual women (see Table S3).

More research is needed to further investigate similarities and differences in age effects on partner preferences based on sexual orientation. In their recent overview, Frederick et al. (2023) concluded that there is a tremendous variety in mating strategies across individuals. They advocate that environmental, social, ecological, and evolutionary factors need to be considered when investigating the relationship between sexual orientation and the mating strategies of people across diverse sexual and gender identities. Comparing the patterns observed for lesbian and bisexual women with the patterns observed in our main analyses can provide the basis for further research investigating partner preferences in nonheterosexual individuals.

### Strengths

To our knowledge, this study was the first to address the question of age effects on partner preferences in a large, international sample of single women. The analysis sample showed a comparably large age range (18‒67), and women were from diverse origins (147 different countries, 10 different languages). By testing preregistered hypotheses with a predefined threshold indicating a substantial effect based on effect sizes previously reported in the literature, we not only were able to detect potential age effects on partner preferences but are also confident to say that, for most preference outcomes, no or only negligible age effects exist. Yet, the inclusion of preference for a partner’s parenting intention added another important aspect missing from previous studies investigating age effects. In addition, employing extensive robustness checks and exploratory analyses, we were able to probe robustness of the attained effects and their generalizability to women with nonheterosexual orientations (namely, lesbian and bisexual women).

### Limitations and Future Research

The current study had some limitations that should be considered when interpreting its results. First, participants were mainly recruited using the menstrual cycle tracking app Clue. Therefore, participants might differ from the broader population and from those in previous studies that have looked at age effects. One of those differences is the age structure of the sample itself. To gauge the generalizability of results, it is especially important to define and describe the age groups in the study’s focus. While the oldest women in the current study were around 67 years of age, other studies defined older participants as 65 years and older (e.g., McIntosh et al., 2011) or even 76 and older (Alterovitz & Mendelsohn, 2011). A number of important life events can happen after the age of 67 (e.g., retirement, health degradation), and these events or their anticipation might shape partner preferences. In addition, an evolutionary perspective suggests that at a certain age there might be a switch from mating and parenting effort to grandparenting effort (Coall et al., 2018). Hence, a potential partner could be seen in a grandparenting role and the partner preference attributes deemed to be most attractive might vary accordingly. Therefore, some of the age effects found here as well as their absence for multiple partner preferences might only hold true within the age range observed in the current study (18‒67). Future research investigating potential age effects on partner preferences should therefore try to take a more extended perspective on the human life span and also strive to sample participants older than 70.

Second, our sample mainly consisted of women from western(ized), industrialized countries (see Fig. 2). Despite aiming for a large and heterogeneous sample, the proportions of women living in African (*n* = 147, 1%) and Asian (n = 792, 5%) countries were very small. Therefore, the results cannot be generalized to all women, and the fact that most of our participants were exposed to and shared “European-westernized” cultural norms should be taken into account.

Third, our cross-sectional study cannot disentangle age effects in the sense of development from potential cohort effects. Future research implementing longitudinal designs could help to address this and could further zoom in on life events (e.g., childbirth) potentially altering partner preferences within individuals. From a methodological perspective, we further urge researchers investigating any kind of potential U-shaped relationship to make use of the two-lines approach by Simonsohn (2018) to determine the tipping point.

Fourth, because of space restrictions in the Ideal Partner Survey, we resorted to a categorical measure of sexual orientation where women had to self-classify. However, this measurement of sexual orientation likely is an oversimplification. In particular, Beaulieu-Prévost and Fortin (2015) highlight the importance of using multiple measures to capture heterogeneity inside artificially classified groups of sexual orientations. Therefore, our results from the exploratory analyses into potential age effects on partner preferences in self-classified lesbian and bisexual women and comparisons with the main analyses based on heterosexual women should only be interpreted with caution. Future research could implement a more fine-grained measure of sexual orientation focusing on attractions, behaviors, and self-identification (Institute of Medicine, 2011) to see how these different domains of sexual orientation potentially influence the link between age and partner preferences. For a comprehensive treatment of how to assess sexual orientation and sexual identity, see the chapters on “Identity and Orientation” in Milhausen et al. (2019).

## Conclusion

The current study suggests that age plays a negligible role in shaping many partner preference dimensions. Nevertheless, age was linked to specific preferences. We found evidence for a positive link between age and preference for confidence-assertiveness, and analyses suggested a linear and a quadratic link between age and preference for intention to become a parent. In addition, higher age was linked to an increased acceptance for younger partners but constant levels of acceptance for older partners, resulting in a broader age range deemed acceptable with increasing age. While cohort effects could not be ruled out on the basis of correlational data, future research based on longitudinal designs may help to discern how development across age shapes partner preferences and associated choices.

### Electronic Supplementary Material

Below is the link to the electronic supplementary material.

Supplementary Material 1

## Data Availability

This study is part of a larger project, the Ideal Partner Survey, for which supporting information can be found on the Open Science Framework (OSF): https://osf.io/wkzng/. Our hypotheses and methods were preregistered on the OSF: https://osf.io/qe3dr/. The authors of the preregistration (i.e., first and last author of this manuscript) did not have access to the data prior to uploading the preregistration; the data sharing agreement is available at: https://osf.io/nvk5r/. Since we cannot share the data publicly, we uploaded a synthetic dataset created with the R-package synthpop (Nowok et al., 2016) based on the sample included in our analyses to the OSF: https://osf.io/qkr9a/. The analysis code is uploaded as part of the accompanying project on the OSF: https://osf.io/mxf9p/

## Footnotes

1 Even though evolutionary theories explaining these differences are built around the concept of sex as a biological factor, the existing literature often used gender as a sociological factor to distinguish between men and women.

2 The preregistration did not include hypotheses for linear effects of age on importance of shared preference for number of children or preferred level of partner’s intention to become a parent for models including a quadratic effect of age. For the sake of completeness and accuracy, we include specific hypotheses about linear effects that are in keeping with our linear hypotheses H6a and H6b.

3 Italian was not mentioned in the preregistration.

4 Setting age values exceeding 100 as missing was not mentioned in the preregistration.

5 The small correlation among the kind-supportive items is probably due to ceiling effects. The means (standard deviations) for the items were *M*_kind−importance_ = 5.50 (*SD*_kind−importance_ = 0.81), *M*_supportive−importance_ = 5.32 (*SD*_supportive−importance_ = 0.86), *M*_kind−level_ = 5.28 (*SD*_kind−level_ = 0.85), and *M*_supportive−level_ = 5.03 (*SD*_supportivelevel_ = 0.90) on a scale ranging from 0 to 6. Ceiling effects for warmth-trustworthiness (i.e., kindness-supportiveness) have been reported in previous preference studies (e.g., Fletcher et al., 1999; Gerlach et al., 2019).

6 Setting minimum and maximum ideal age values exceeding 100 as missing was not mentioned in the preregistration.

7 Due to a technical error the answer to this seriousness question was missing in 1,952 cases. The preregistration did not mention what to do in case of missing answers. Since the technical error was random, we decided to keep the individuals for our main analyses. In addition, we performed extensive robustness checks.

8 In the preregistration we mentioned that we would include a random intercept and a random slope for country, but none of the main models converged. Therefore, all our analyses were performed without a random slope but with a random intercept (this applies to all exploratory analyses and robustness analyses with a random intercept for language instead of a random intercept for country).
