# Supplementary material for: The Link Between Age and Partner Preferences in a Large, International Sample of Single Women
Source: Hum Nat. 2023 Sep 26;34(4):539–68. doi: 10.1007/s12110-023-09460-4 (PMC10739319; doi:10.1007/s12110-023-09460-4)
Supplement: Supplementary file 1 — Supplementary Material 1 [file 12110_2023_9460_MOESM1_ESM.pdf]

Supplement for "The Link Between Age and Partner Preferences in a Large, International Sample of Single Women"

***Human Nature 34*(3), 2023, https://doi.org/10.1007/s12110-023-09460-4**

Laura J. Botzet (0000-0001-5765-8354)[a,b], **Amanda Shea** (0000-0002-8846-8841)[c], **Virginia J. Vitzthum** (0000-0002-6765-4557)[c,d,e], **Anna Druet** (0000-0002-3340-5210)[c], **Maddie Sheesley**[c], **and Tanja M. Gerlach**[f,a,b]

[a] Department for Biological Personality Psychology, University of Goettingen, Germany
[b] Leibniz Science Campus Primate Cognition, Deutsches Primatenzentrum GmbH, Germany;
[c] Clue by BioWink GmbH, Germany;
[d] Evolutionary Anthropology Laboratory, Indiana University, USA;
[e] Department of Anthropology, Indiana University, USA;
[f] School of Psychology, Queen's University Belfast, United Kingdom

Corresponding Author:
Laura J. Botzet, E-mail: botzet@uni-goettingen.de

This study is part of a larger project, the *Ideal Partner Survey,* for which supporting information can be found on the Open Science Framework (OSF): https://osf.io/wkzng/

Deviations from Preregistration

Our hypotheses and methods were preregistered on the Open Science Framework: https://osf.io/qe3dr/. We deviated in our preregistration in several instances. These are mentioned as footnotes throughout the main manuscript and described in more detail in Table S1.

Table S1

Deviations from our original preregistration https://osf.io/qe3dr/

Footnote	Components in preregistration	Details of and rationale for the deviation	How might the deviation affect the results?
2	Hypotheses on parenting intention	The preregistration did not include hypotheses for linear effects of age on importance of shared preference for number of children or preferred level of partner's intention to become a parent for models including a quadratic effect of age. For the sake of completeness and accuracy we include specific hypotheses about linear effects that are in line with linear hypotheses H6a) and H6b).	Hypotheses are in line with the other hypotheses that were specified in the preregistration.
3	Languages of the Ideal Partner Survey	The preregistration reports only nine language versions. Italian is erroneously missing from this list.<br>1,265 of 17,254 women in our main sample filled out the survey in Italian (221 of 3,553 in our exploratory analyses).	Excluding these women from our analyses would have greatly reduced the sample size and seemed disproportionate given that we did not expect associations in Italian speaking women to differ from the rest of the sample.

Table S1 (continued)
Deviations from our original preregistration https://osf.io/qe3dr/

Footnote	Components in preregistration	Details of and rationale for the deviation	How might the deviation affect the results?
4, 6	Range of age values in the Ideal Partner Survey	Values for own age as well as minimum and maximum ideal age were not restricted to a certain range.	All age values exceeding 100 were set to missing. This affected 15 women in the raw dataset and 5 women in the main sample. Setting minimum (maximum) ideal age values > 100 to missing affected 2 women in the raw dataset and nobody in the main sample (1 woman in the raw dataset and nobody in the main sample). We do not think that this deviation affected the reported results.
7	Seriousness question	Due to a technical error the answer to the seriousness question was missing in 1,952 cases. The preregistration did not mention what to do in case of missing answers. Since the technical error was random we decided to keep the 1,952 individuals for our main analyses. In addition, we performed extensive robustness checks.	None of the robustness checks led to diverging results, therefore the decision to include women with missing answers to the seriousness question likely did not affect results. Both approaches (potentially including unserious women in the main analyses and potentially excluding serious women in the robustness analyses) may have lowered the ability to detect small effects (the first approach by introducing random noise, the second approach by reducing sample size).

Table S1 (continued)
Deviations from our original preregistration https://osf.io/qe3dr/

Footnote	Components in preregistration	Details of and rationale for the deviation	How might the deviation affect the results?
8	Model convergence	The preregistration states that we would include a random intercept and slope for country. As all main models did not converge when including a random slope for country, all analyses (including robustness and exploratory analyses) were performed only with a random intercept for country/ language. This procedure was described in the preregistration but to keep the main article short and precise we did not include the detailed model convergence procedure in the main manuscript.	As this is not a real deviation from the preregistration but rather a shortened description of the procedure with model convergence it did not affect the results.

Note. This deviation form was inspired by the Preregistration Planning and Deviation Documentation (PPDD) template: https://osf.io/ywrqe/ (Van't Veer et al., 2019).

Detailed Description of Countries

Table S2
Country distribution for all women (n = 17,254)

Country	n	Percentage	Country	n	Percentage
Afghanistan	1	0.01%	Côte d'Ivoire	5	0.03%
Albania	3	0.02%	Croatia	7	0.04%
Algeria	8	0.05%	Cuba	3	0.02%
Andorra	10	0.06%	Czechia	12	0.07%
Antigua and Barbuda	3	0.02%	Denmark	459	2.66%
Argentina	331	1.92%	Dominica	2	0.01%
Armenia	2	0.01%	Dominican Republic	57	0.33%
Aruba	1	0.01%	East Timor (Timor-Leste)	1	0.01%
Australia	173	1.00%	Ecuador	138	0.80%
Austria	244	1.41%	Egypt	5	0.03%
Bahamas	1	0.01%	El Salvador	43	0.25%
Bahrain	3	0.02%	Estonia	13	0.08%
Barbados	2	0.01%	Ethiopia	1	0.01%
Belarus	13	0.08%	Fiji	1	0.01%
Belgium	137	0.79%	Finland	35	0.20%
Belize	2	0.01%	France	2,716	15.74%
Benin	2	0.01%	Georgia	5	0.03%
Bolivia	27	0.16%	Germany	2,181	12.64%
Bosnia and Herzegovina	6	0.03%	Ghana	3	0.02%
Botswana	1	0.01%	Greece	5	0.03%
Brazil	1,062	6.16%	Grenada	4	0.02%
Bulgaria	10	0.06%	Guatemala	83	0.48%
Burma	1	0.01%	Guinea-Bissau	1	0.01%
Cameroon	4	0.02%	Guyana	3	0.02%
Canada	439	2.54%	Haiti	5	0.03%
Central African Republic	2	0.01%	Honduras	24	0.14%
Chile	200	1.16%	Hong Kong	10	0.06%
China	106	0.61%	Hungary	16	0.09%
Colombia	545	3.16%	Iceland	5	0.03%
Costa Rica	71	0.41%	India	58	0.34%

Table S2 (continued)
Country distribution for all women (n = 17,254)

Country	n	Percentage	Country	n	Percentage
Indonesia	22	0.13%	Montenegro	1	0.01%
Iran	11	0.06%	Morocco	22	0.13%
Iraq	1	0.01%	Namibia	4	0.02%
Ireland	73	0.42%	Nepal	1	0.01%
Israel	15	0.09%	Netherlands	58	0.34%
Italy	1,230	7.13%	New Zealand	48	0.28%
Jamaica	8	0.05%	Nicaragua	17	0.10%
Japan	351	2.03%	Nigeria	8	0.05%
Jordan	2	0.01%	Norway	16	0.09%
Kazakhstan	10	0.06%	Oman	1	0.01%
Kenya	9	0.05%	Pakistan	10	0.06%
Kuwait	1	0.01%	Palestinia	2	0.01%
Kyrgyzstan	2	0.01%	Panama	34	0.20%
Latvia	10	0.06%	Paraguay	16	0.09%
Lebanon	4	0.02%	Peru	164	0.95%
Liechtenstein	1	0.01%	Philippines	46	0.27%
Lithuania	6	0.03%	Poland	7	0.04%
Luxembourg	16	0.09%	Portugal	71	0.41%
Macedonia	1	0.01%	Qatar	3	0.02%
Madagascar	2	0.01%	Romania	27	0.16%
Malaysia	25	0.14%	Russia	197	1.14%
Maldives	1	0.01%	Saint Lucia	2	0.01%
Mali	2	0.01%	Saint Vincent and the Grenadines	1	0.01%
Malta	3	0.02%	San Marino	1	0.01%
Marshall Islands	1	0.01%	Saudi Arabia	11	0.06%
Mauritania	1	0.01%	Senegal	7	0.04%
Mauritius	4	0.02%	Serbia	7	0.04%
Mexico	1,595	9.24%	Singapore	31	0.18%
Micronesia	1	0.01%	Sint Maarten	2	0.01%
Monaco	1	0.01%	Slovakia	3	0.02%

Table S2 (continued)

Country distribution for all women (n = 17,254)

Country	n	Percentage	Country	n	Percentage
Slovenia	4	0.02%	Tunisia	11	0.06%
South Africa	39	0.23%	Turkey	10	0.06%
South Korea	5	0.03%	Turkmenistan	2	0.01%
South Sudan	1	0.01%	Tuvalu	1	0.01%
Spain	755	4.38%	Uganda	1	0.01%
Sri Lanka	3	0.02%	Ukraine	24	0.14%
Swaziland	1	0.01%	United Arab Emirates	17	0.10%
Sweden	38	0.22%	United Kingdom	635	3.68%
Switzerland	361	2.09%	United States of America	1,637	9.49%
Syria	1	0.01%	Uruguay	37	0.21%
Taiwan	7	0.04%	Venezuela	103	0.60%
Tanzania	1	0.01%	Vietnam	4	0.02%
Thailand	6	0.03%	Zimbabwe	2	0.01%
Trinidad and Tobago	10	0.06%			

Scatterplots for Age Effects on Partner Preferences

Fig. S1

Partner preferences by own age controlled for a random intercept for country

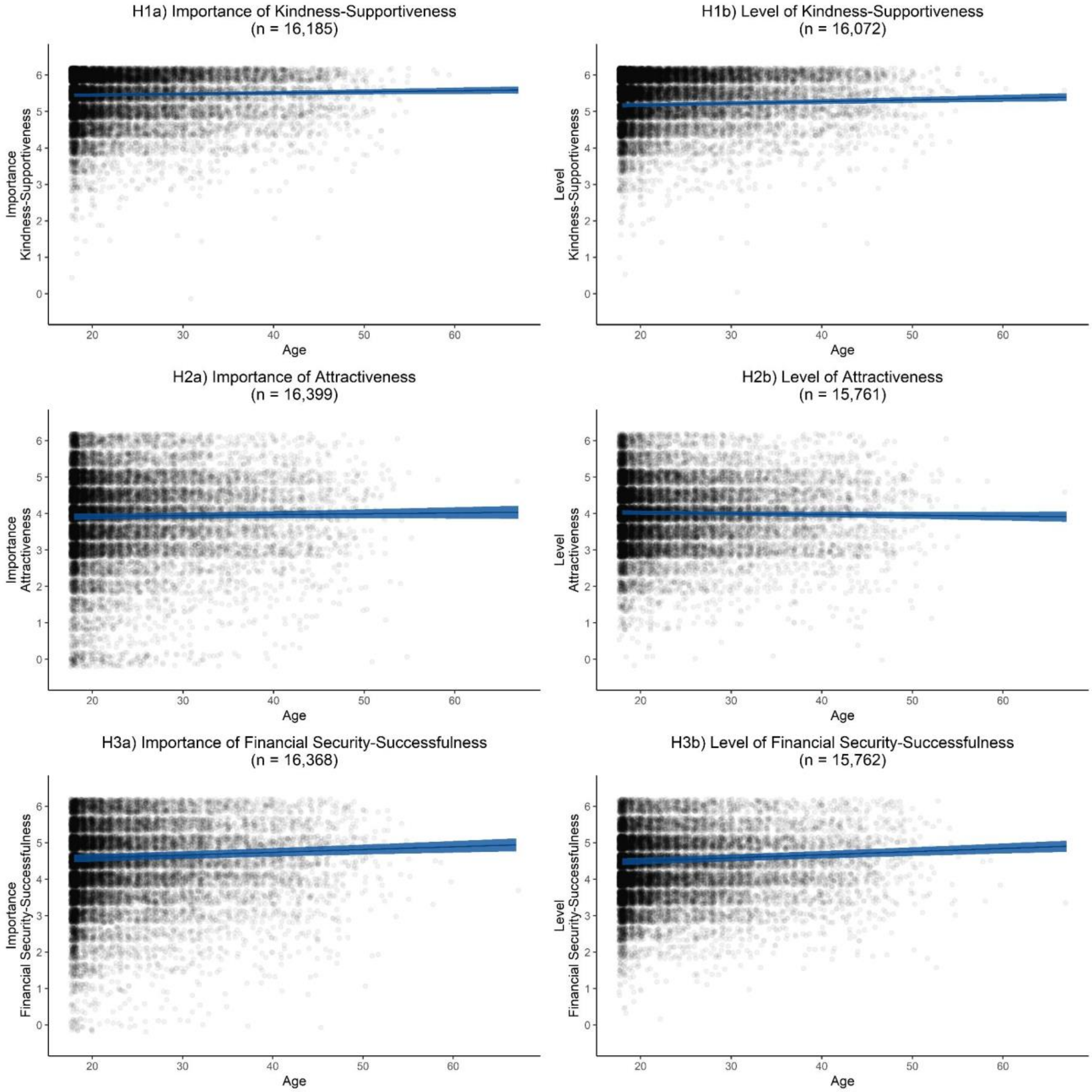

Fig. S1 (continued)

Partner preferences by own age controlled for a random intercept for country

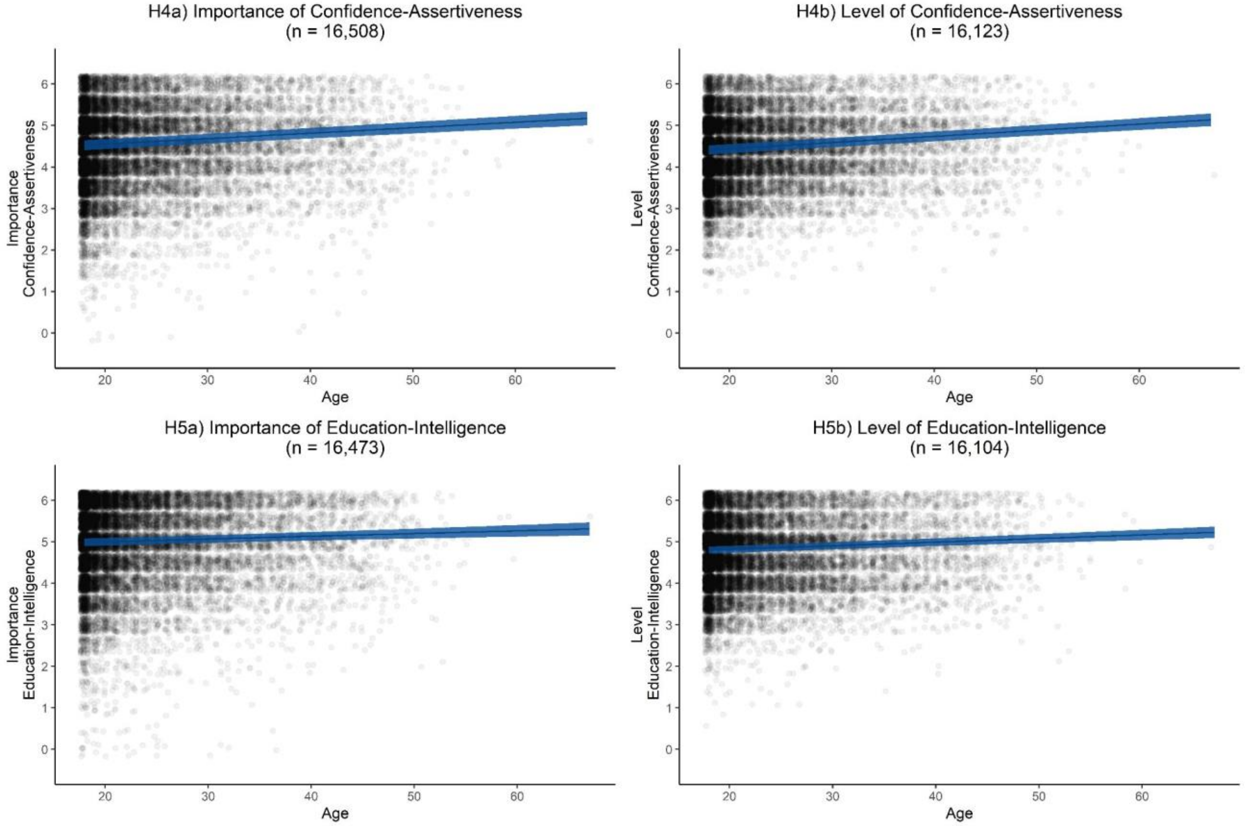

Note. Ideal partner preferences include preferences for kindness-supportiveness (H1a) & H1b)), attractiveness (H2a) & H2b)), financial security-successfulness (H3a) & H3b)), confidence-assertiveness (H4a) & H4b)), and education-intelligence (H5a) & H5b)). Graphs display importance ratings (indicated by a)) and preferred level (indicated by b)) of the respective partner preferences. Blue areas represent 99.5% confidence intervals.

Two-Lines Approach by Simonsohn (2018)

Fig. S2

Importance rating for shared preference for number of children by age applying the two-lines approach by Simonsohn (2018)

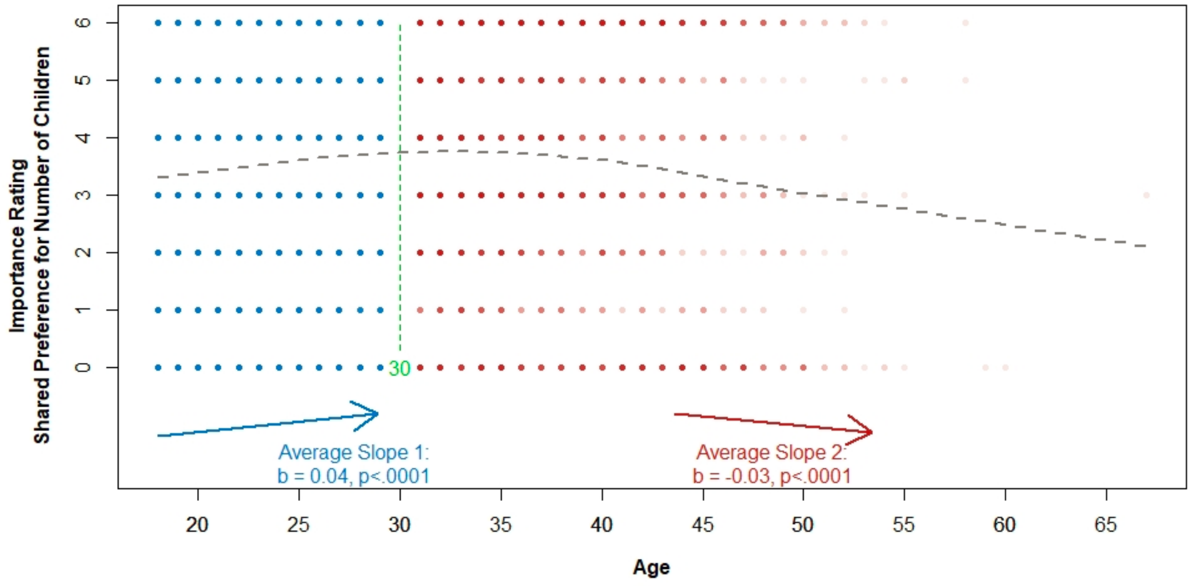

Note. Graph and underlying analysis are based on R code provided at http://webstimate.org/twolines/. Visuals are slightly adapted to enhance readability.

Fig. S3

Preferred level of partner's intention to become a parent by age applying the two-lines approach by Simonsohn (2018)

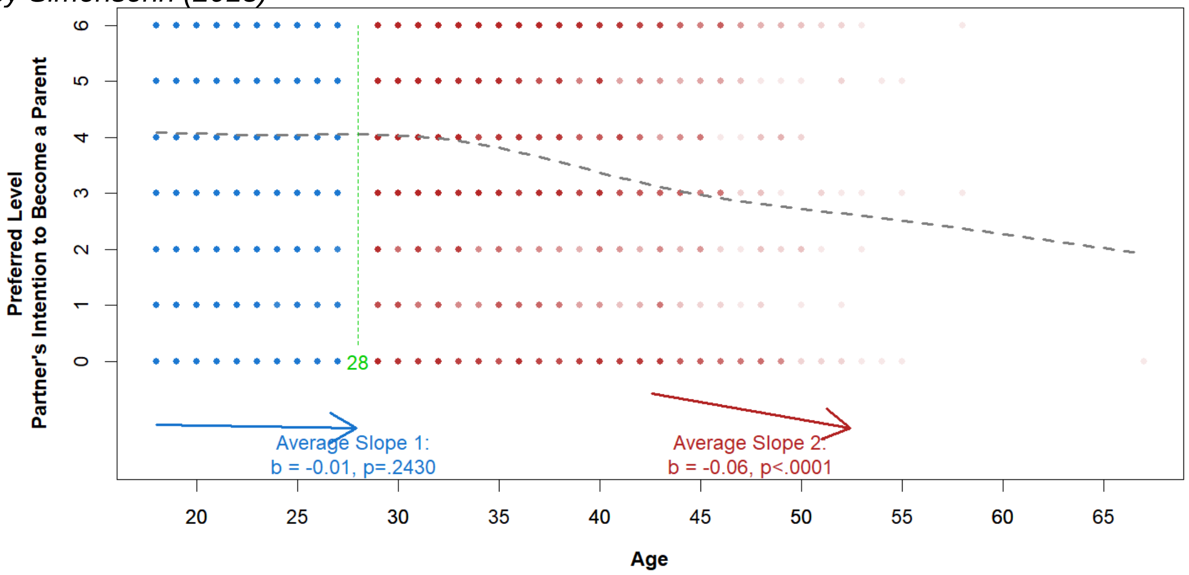

Note. Graph and underlying analysis are based on R code provided at http://webstimate.org/twolines/. Visuals are slightly adapted to enhance readability.

Exploratory Analyses

Exclusion Steps

For our exploratory analyses for lesbian and bisexual women we excluded the following participants: (1) all participants who did not identify as women; (2) all participants who did not identify as lesbian/gay/homosexual or bisexual/pansexual; (3) all participants who indicated being in a relationship or where it was not certain if participants were currently single; (4) all participants who did not answer the survey seriously or chose not to answer the seriousness question[1]. This led to sample sizes of 467 women identifying as lesbian and 3,085 women identifying as bisexual. Exclusion steps for our exploratory analyses are illustrated in Fig. S4.

[1] Due to a technical error the answer to this question was missing in 369 cases in the sample of bisexual women and in 41 cases in the sample of lesbian women. The preregistration did not mention what to do in case of missing answers. Since the technical error was random we decided to keep the individuals for our analyses. In addition, we performed extensive robustness checks. See footnote 6 in Table S1 for further information.

Fig. S4
Exclusion steps for exploratory analyses.

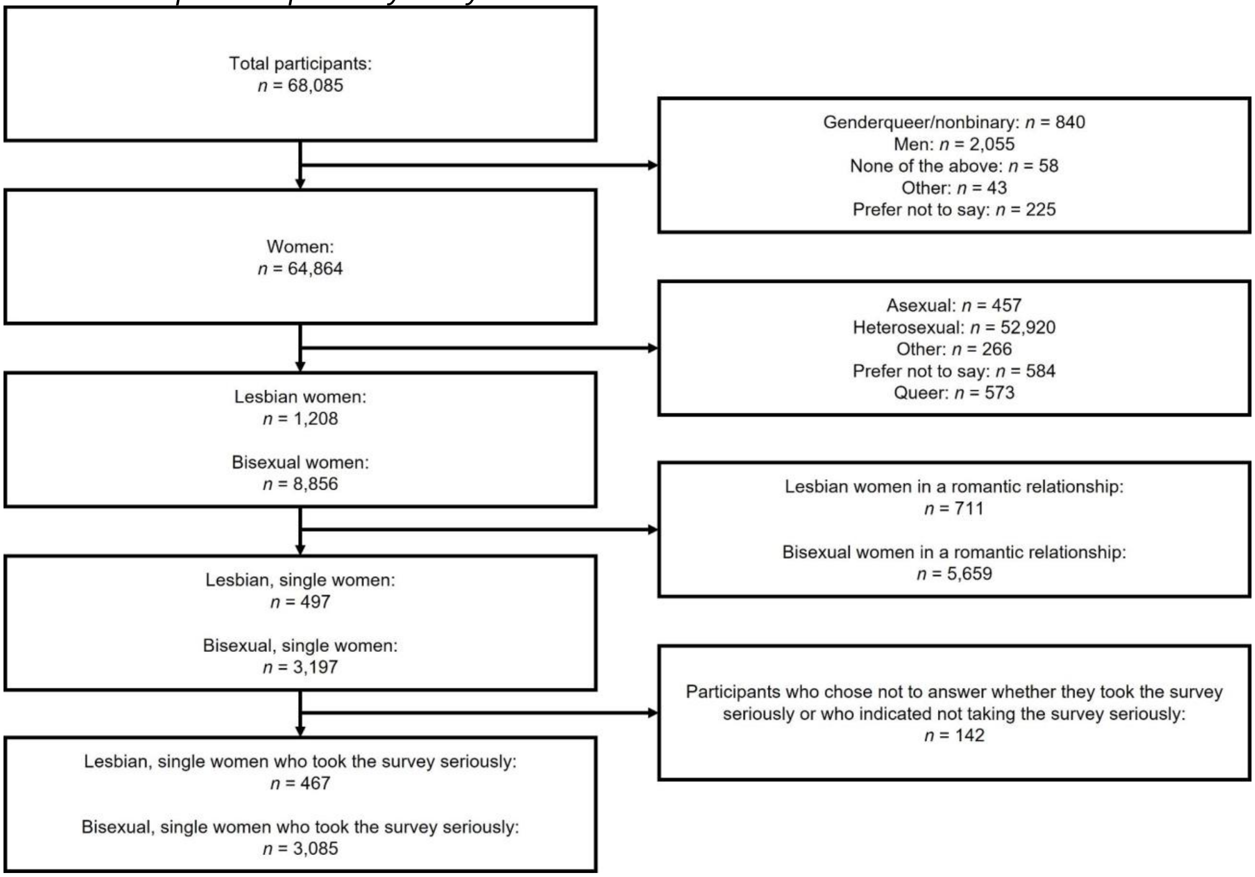

Table S3

Means (standard deviations) and Cohen's ds *for age, partner preferences, and preference for ideal age-range for heterosexual, lesbian, and bisexual women*

	Groups			Comparisons Cohen's *d* [CI]		
**Variable**	**(1) Heterosexual (*n* = 17,254)**	**(2) Lesbian (*n* = 467)**	**(3) Bisexual (*n* = 3,085)**	**(1) Heterosexual compared to (2) Lesbian**	**(1) Heterosexual compared to (3) Bisexual**	**(2) Lesbian compared to (3) Bisexual**
Own Age	23.58 (6.92)	21.99 (5.48)	21.34 (4.78)	**0.26 [0.16, 0.34]**	**0.38 [0.34, 0.41]**	**0.13 [0.03, 0.23]**
Kindness-Supportiveness (I)	5.41 (0.66)	5.40 (0.71)	5.37 (0.71)	0.01 [-0.08, 0.11]	**0.06 [0.02, 0.10]**	0.05 [-0.06, 0.14]
Kindness-Supportiveness (L)	5.15 (0.70)	5.08 (0.73)	5.04 (0.72)	**0.10 [0.01, 0.20]**	**0.16 [0.12, 0.20]**	0.05 [-0.04, 0.15]
Attractiveness (I)	3.96 (1.16)	3.64 (1.40)	3.63 (1.27)	**0.24 [0.14, 0.35]**	**0.27 [0.23, 0.31]**	0.01 [-0.09, 0.11]
Attractiveness (L)	4.04 (0.92)	3.87 (0.96)	3.81 (0.93)	**0.17 [0.08, 0.28]**	**0.24 [0.20, 0.28]**	0.06 [-0.04, 0.17]
Financially Secure-Successful (I)	4.41 (1.08)	3.86 (1.24)	4.10 (1.21)	**0.48 [0.38, 0.57]**	**0.28 [0.24, 0.32]**	**-0.19 [-0.29, -0.09]**
Financially Secure-Successful (L)	4.35 (0.90)	4.00 (0.87)	4.10 (0.92)	**0.40 [0.30, 0.50]**	**0.27 [0.23, 0.31]**	**-0.12 [-0.22, -0.02]**
Confidence-Assertiveness (I)	4.50 (0.94)	4.00 (1.09)	4.24 (1.03)	**0.49 [0.40, 0.59]**	**0.26 [0.23, 0.30]**	**-0.23 [-0.33, -0.13]**
Confidence-Assertiveness (L)	4.40 (0.85)	4.03 (0.86)	4.19 (0.85)	**0.43 [0.33, 0.53]**	**0.24 [0.21, 0.28]**	**-0.19 [-0.29, -0.08]**
Education-Intelligence (I)	4.93 (0.96)	4.64 (1.19)	4.81 (1.06)	**0.27 [0.17, 0.36]**	**0.12 [0.09, 0.16]**	**-0.15 [-0.25, -0.04]**
Education-Intelligence (L)	4.73 (0.83)	4.50 (0.93)	4.59 (0.85)	**0.26 [0.15, 0.37]**	**0.16 [0.12, 0.20]**	-0.10 [-0.21, 0.00]
Importance of Shared Preference for Number of Children	3.47 (1.96)	3.30 (2.05)	3.22 (2.14)	0.08 [-0.01, 0.19]	**0.13 [0.09, 0.17]**	0.04 [-0.05, 0.15]
Preferred Level of Partner's Intention to Become a Parent	4.00 (1.82)	3.00 (1.93)	3.10 (1.95)	**0.53 [0.44, 0.64]**	**0.48 [0.43, 0.52]**	-0.06 [-0.17, 0.05]
Ideal Age-Range	8.12 (6.23)	9.03 (7.66)	9.32 (6.41)	**-0.13 [-0.24, -0.01]**	**-0.19 [-0.23, -0.15]**	-0.04 [-0.16, 0.09]
Youngest Age Deemed Acceptable Relative to Own Age	-1.21 (5.37)	-1.95 (3.92)	-0.87 (3.85)	**-0.16 [-0.25, -0.08]**	**0.07 [0.03, 0.11]**	**0.28 [0.16, 0.38]**
Oldest Age Deemed Acceptable Relative to Own Age	6.91 (7.43)	7.08 (7.43)	8.45 (6.91)	-0.02 [-0.13, 0.09]	**-0.21 [-0.26, -0.17]**	**-0.19 [-0.33, -0.06]**

Note. Bold Cohen's *d*s indicate that groups differ significantly (p < .05). CI = 95% confidence interval.

Results Exploratory Analyses Based on Lesbian Women

Table S4

Main results for age effects on partner preferences including preferences for kindness-supportiveness, attractiveness, financial security-successfulness, confidence-assertiveness, and education-intelligence in the sample of lesbian women

Hypothesis	Outcome		*n*	Countries	Linear Age Effect *b [CI]*	Linear Age Effect *β [CI]*	*p*
H1a)	Kindness-supportiveness	Importance	449	48	0.00 [-0.01, 0.02]	0.03 [-0.10, 0.16]	.53
H1b)		Level	447	48	0.00 [-0.01, 0.02]	0.03 [-0.10, 0.16]	.54
H2a)	Attractiveness	Importance	451	48	0.04 [0.01, 0.08]	0.16 [0.03, 0.30]	< .001
H2b)		Level	416	48	0.04 [0.02, 0.06]	0.22 [0.09, 0.36]	< .001
H3a)	Financially secure-successful	Importance	451	48	0.02 [-0.01, 0.05]	0.09 [-0.03, 0.22]	.04
H3b)		Level	417	48	0.02 [-0.01, 0.05]	0.14 [0.01, 0.28]	.002
H4a)	Confidence-assertiveness	Importance	454	48	0.03 [0.00, 0.05]	0.14 [0.02, 0.26]	.002
H4b)		Level	439	47	0.03 [0.01, 0.05]	0.20 [0.08, 0.33]	< .001
H5a)	Education-intelligence	Importance	453	48	0.02 [-0.01, 0.05]	0.09 [-0.05, 0.22]	.07
H5b)		Level	432	48	0.02 [0.00, 0.05]	0.14 [0.00, 0.27]	.004

Note. All analyses included a linear effect of age as a predictor and a random intercept for country. Sample size and number of countries differ for reasons explained in detail in the section “Sample Characteristics” of the main manuscript. For all hypotheses the raw and the standardized beta coefficient for the linear effect of age are displayed. The effect of age never reached substantiality ($p < .005$ and $|\beta| > 0.10$ with *CI*s excluding ±0.10).

b = raw beta coefficient, *β* = standardized beta coefficient, *CI* = 99.5% confidence interval.

Fig. S5

Effect size estimates of linear age effects on partner preferences for main analyses and three robustness analyses in the sample of lesbian women

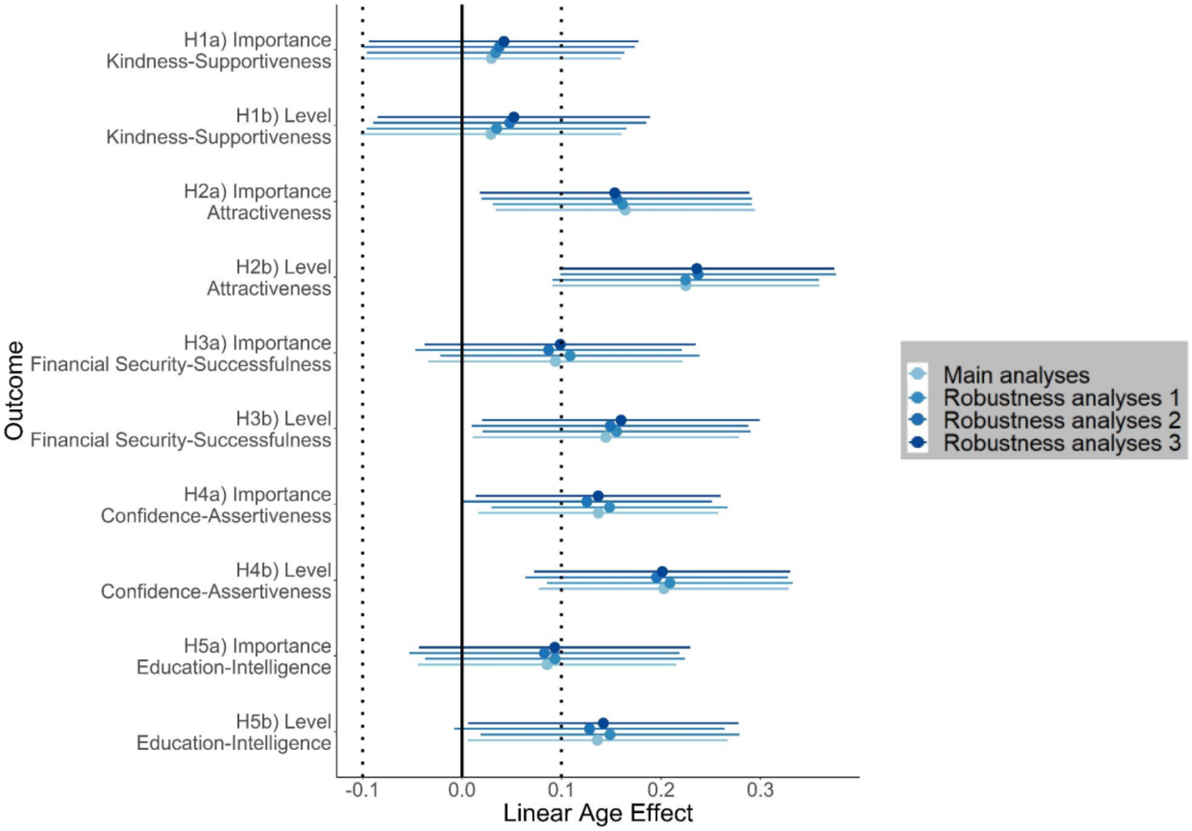

Note. Ideal partner preferences include preferences for kindness-supportiveness (H1a) & H1b)), attractiveness (H2a) & H2b)), financial security-successfulness (H3a) & H3b)), confidence-assertiveness (H4a) & H4b)), and education-intelligence (H5a) & H5b)).
Robustness analyses 1: random intercept for language instead of countries; robustness analyses 2: excluding women with missing answers to the seriousness question ($n_{excluded}$ = 41); robustness analyses 3: random intercept for language instead of countries and excluding women with missing answers to the seriousness question ($n_{excluded}$ = 41). Graphs display standardized linear age effects, error bars represent 99.5% confidence intervals. Dotted lines indicate the inference criteria for substantial effects *(*$p < .005$ and $|\beta| > 0.10$ with *CI*s excluding ±0.10*).*

Table S5

Main results for age effects on parenting intention in the sample of lesbian women

Hypothesis	Outcome	*n*	Countries	Predictor	Age Effect *b [CI]*	Age Effect *β [CI]*	*p*
H6a)	Importance of shared preference for number of children	456	48	linear	0.01 [-0.04, 0.06]	0.02 [-0.11, 0.15]	.65
H6b)	Preferred level of partner's intention to become a parent	399	45	linear	0.01 [-0.04, 0.07]	0.03 [-0.11, 0.18]	.49
H7a)	Importance of shared preference for number of children	456	48	linear	0.19 [-0.19, 0.58]	0.50 [-0.52, 1.52]	.17
				quadratic	-0.00 [-0.01, 0.00]	-0.49 [-1.51, 0.53]	.18
H7b)	Preferred level of partner's intention to become a parent	399	45	linear	-0.19 [-0.61, 0.22]	-0.51 [-1.61, 0.59]	.19
				quadratic	0.00 [-0.00, 0.01]	0.55 [-0.55, 1.65]	.16

Note. Models for hypotheses H6a and H6b included age as a linear predictor and a random intercept for country. Models for hypotheses H7a and H7b included age as a linear and as a quadratic predictor and a random intercept for country. Sample size and number of countries differ for reasons explained in detail in the section "Sample Characteristics" of the main manuscript. For all hypotheses except for H7a and H7b the raw and the standardized beta coefficient for the linear effect of age are displayed. For H7a and H7b the raw and the standardized beta coefficient for the quadratic effect of age are displayed. In the models including only the linear predictor, the effect of age never reached substantiality ($p < .005$ and $|\beta| > 0.10$ with *CI*s excluding ±0.10).
b = raw beta coefficient, *β* = standardized beta coefficient, *CI* = 99.5% confidence interval.

Fig. S6

Effect size estimates of linear and quadratic age effects on preference for parenting intention for main analyses and three robustness analyses in the sample of lesbian women

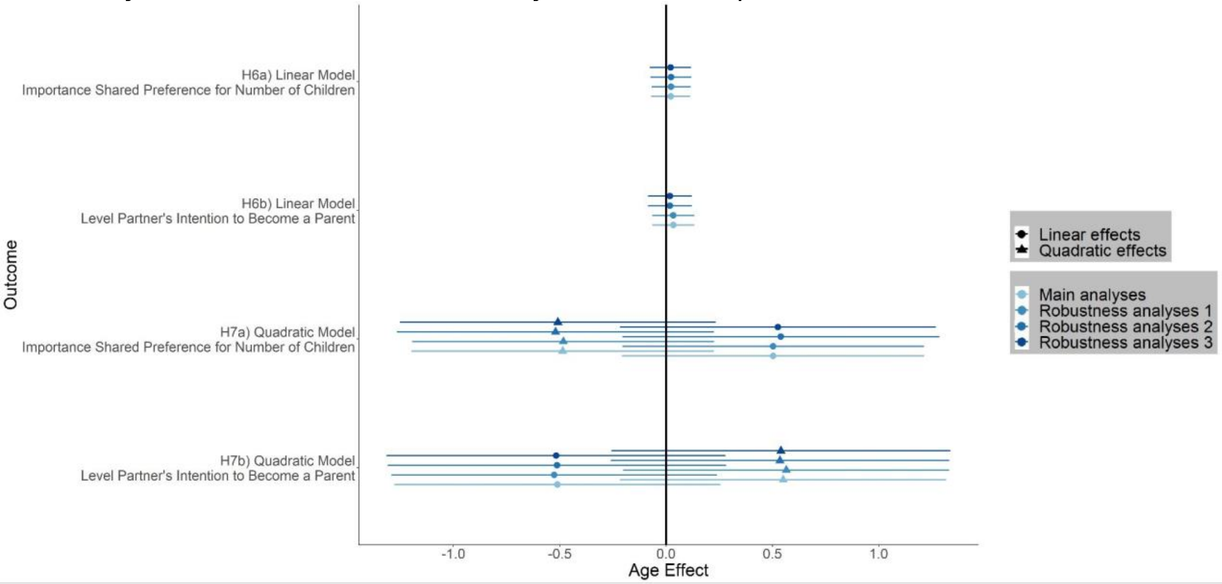

Note. Robustness analyses 1: random intercept for language instead of countries; robustness analyses 2: excluding women with missing answers to the seriousness question ($n_{excluded}$ = 41); robustness analyses 3: random intercept for language instead of countries and excluding women with missing answers to the seriousness question ($n_{excluded}$ = 41). Graphs display standardized linear and quadratic age effects, error bars represent 99.5% confidence intervals. Confidence intervals for effects estimates based on linear models are narrower compared to confidence intervals for effect estimates based on quadratic models because including a quadratic term decreases accuracy of estimates. In the models including only the linear predictor, the effect of age never reached substantiality ($p < .005$ and $|\beta| > 0.10$ with *CI*s excluding ±0.10).

Fig. S7

Preference for parenting intention by a linear and quadratic effect of age controlled for a random intercept for country in the sample of lesbian women

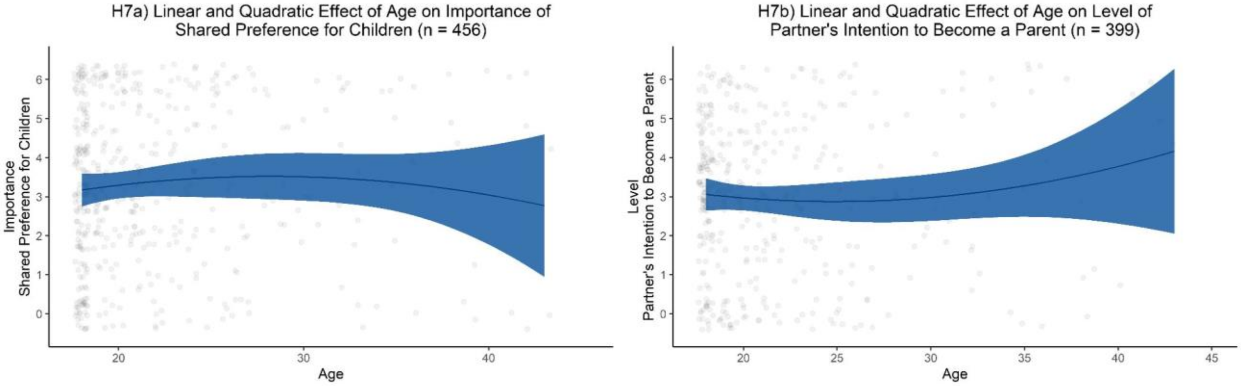

Note. Parenting intention includes importance rating for a partner sharing the preference for number of children (H7a) and preference for the level of partner's intention to become a parent (H7b). Blue areas represent 99.5% confidence intervals.

Table S6

Main results for age effects on ideal age-range in the sample of lesbian women

Hypothesis	Outcome	*n*	Countries	Linear Age Effect *b [CI]*	Linear Age Effect *β [CI]*	*p*
H8)	Age-range deemed acceptable	331	45	**0.51 [0.31, 0.71]**	**0.36 [0.22, 0.51]**	< .001
H9)	Youngest age deemed acceptable	331	45	**0.33 [0.23, 0.42]**	**0.46 [0.33, 0.59]**	< .001
H10)	Oldest age deemed acceptable	331	45	0.18 [-0.02, 0.39]	0.13 [-0.02, 0.28]	.01

Note. All analyses included a linear effect of age as a predictor and a random intercept for country. Sample size and number of countries differ for reasons explained in detail in the section “Sample Characteristics” of the main manuscript. For all hypotheses the raw and the standardized beta coefficient for the linear effect of age are displayed. Bold effect size estimates indicate substantial effects ($p < .005$ and $|\beta| > 0.10$ with *CI*s excluding ±0.10).
b = raw beta coefficient, *β* = standardized beta coefficient, *CI* = 99.5% confidence interval.

Fig. S8

Effect size estimates of linear effects on age-range deemed acceptable and on youngest and oldest age deemed acceptable for main analyses and three robustness analyses in the sample of lesbian women

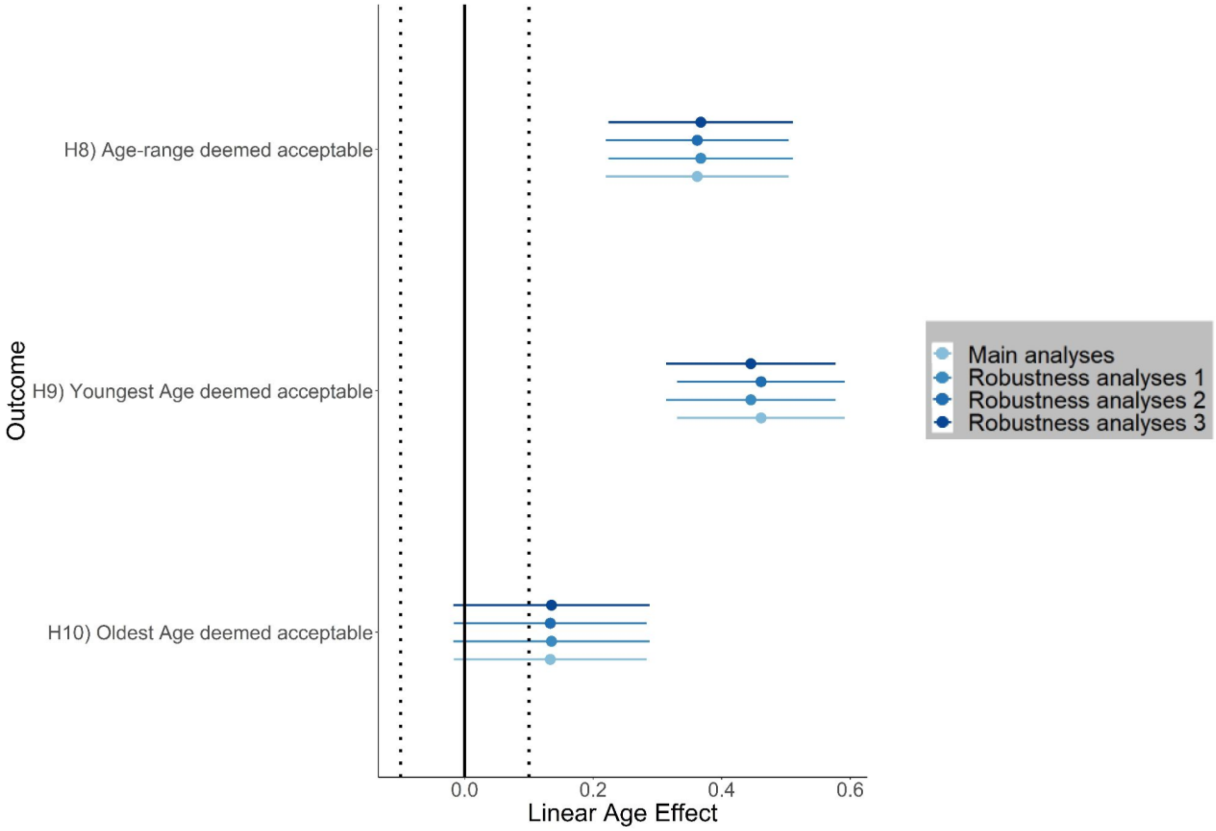

Note. Robustness analyses 1: random intercept for language instead of countries; robustness analyses 2: excluding women with missing answers to the seriousness question ($n_{excluded}$ = 41); robustness analyses 3: random intercept for language instead of countries and excluding women with missing answers to the seriousness question ($n_{excluded}$ = 41). Graphs display standardized linear age effects, error bars represent 99.5% confidence intervals. Dotted lines indicate the inference criteria for substantial effects *(*p < .005 and |β| > 0.10 with *CI*s excluding ±0.10*).*

Fig. S9

Age-range deemed acceptable and youngest and oldest age deemed acceptable by own age in the sample of lesbian women

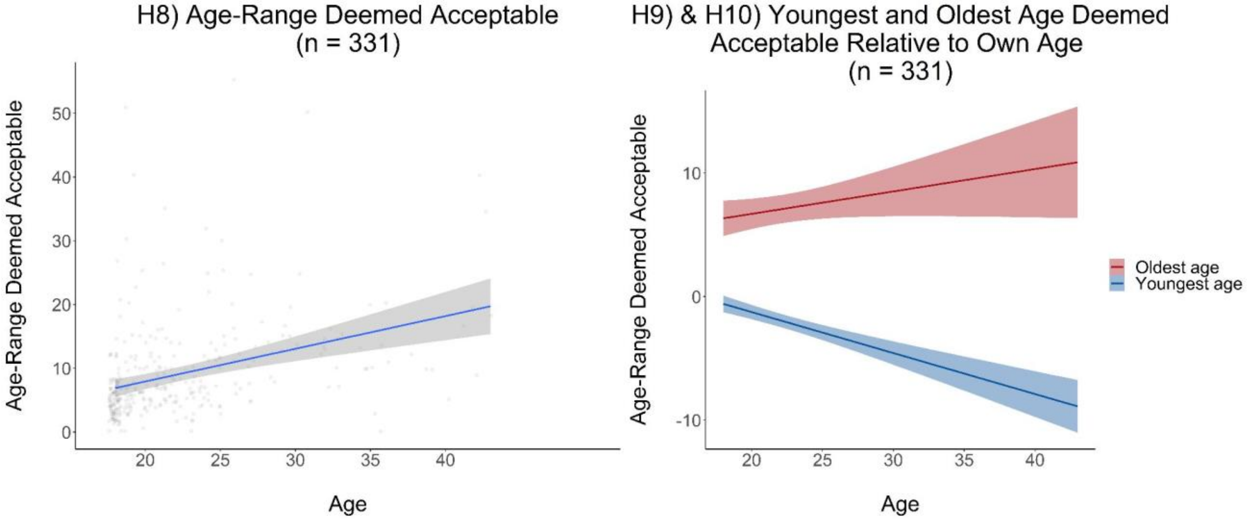

Note. Graphs display means and 99.5% confidence intervals.

Results Exploratory Analyses Based on Bisexual Women

Table S7

Main results for age effects on partner preferences including preferences for kindness-supportiveness, attractiveness, financial security-successfulness, confidence-assertiveness, and education-intelligence in the sample of bisexual women

Hypothesis	Outcome		*n*	Countries	Linear Age Effect *b [CI]*	Linear Age Effect *β [CI]*	*p*
H1a)	Kindness-supportiveness	Importance	2,943	91	0.01 [0.00, 0.02]	0.07 [0.02, 0.12]	< .001
H1b)		Level	2,936	91	0.01 [0.00, 0.02]	0.06 [0.01, 0.11]	< .001
H2a)	Attractiveness	Importance	2,976	92	0.02 [0.01, 0.04]	0.09 [0.04, 0.14]	< .001
H2b)		Level	2,801	91	0.01 [-0.00, 0.02]	0.03 [-0.02, 0.08]	.13
H3a)	Financially secure-successful	Importance	2,968	92	0.01 [-0.01, 0.02]	0.02 [-0.03, 0.07]	.19
H3b)		Level	2,777	91	0.01 [-0.01, 0.02]	0.03 [-0.03, 0.08]	.15
H4a)	Confidence-assertiveness	Importance	2,994	92	0.02 [0.01, 0.03]	0.09 [0.05, 0.14]	< .001
H4b)		Level	2,908	90	0.02 [0.01, 0.02]	0.08 [0.04, 0.13]	< .001
H5a)	Education-intelligence	Importance	2,986	92	0.01 [0.00, 0.03]	0.06 [0.01, 0.11]	< .001
H5b)		Level	2,896	92	0.01 [0.00, 0.02]	0.06 [0.01, 0.11]	< .001

Note. All analyses included a linear effect of age as a predictor and a random intercept for country. Sample size and number of countries differ for reasons explained in detail in the section “Sample Characteristics” of the main manuscript. For all hypotheses the raw and the standardized beta coefficient for the linear effect of age are displayed. The effect of age never reached substantiality ($p < .005$ and $|\beta| > 0.10$ with *CI*s excluding ±0.10).
b = raw beta coefficient, *β* = standardized beta coefficient, *CI* = 99.5% confidence interval.

Fig. S10

Effect size estimates of linear age effects on partner preferences for main analyses and three robustness analyses in the sample of bisexual women

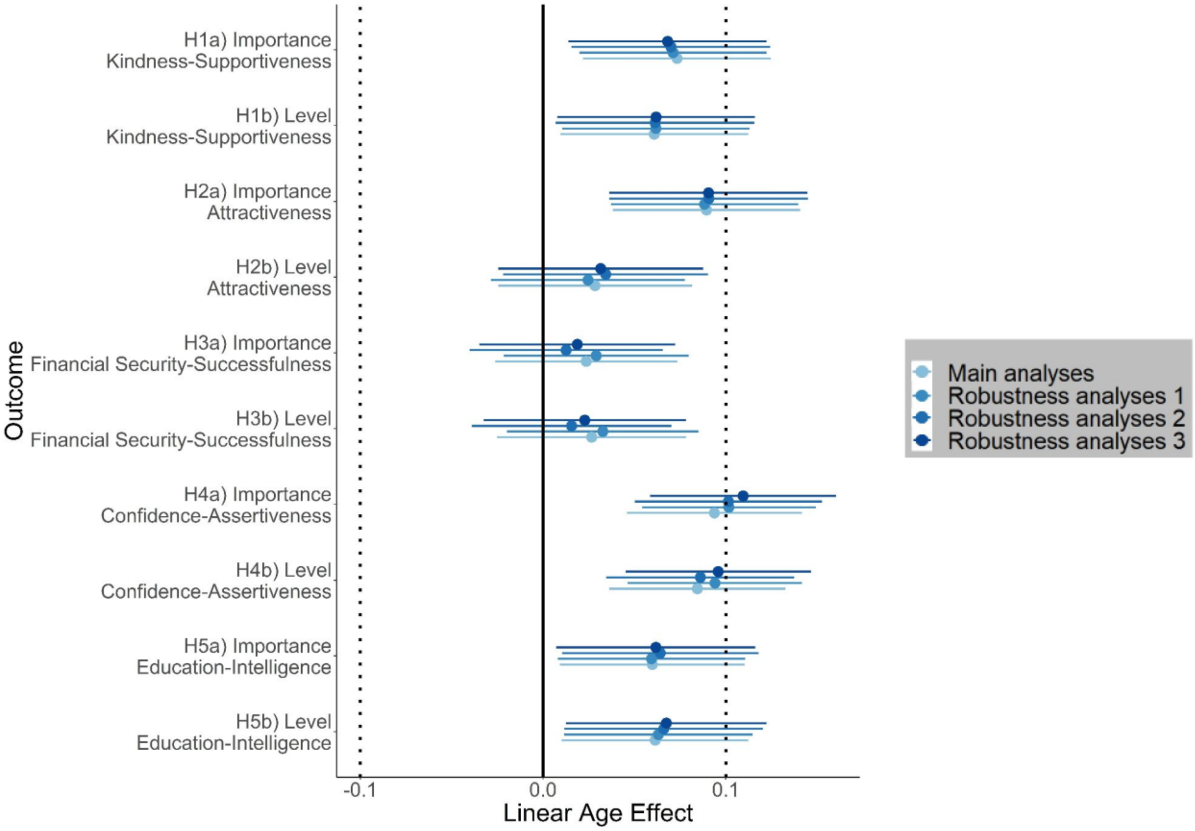

Note. Ideal partner preferences include preferences for kindness-supportiveness (H1a) & H1b)), attractiveness (H2a) & H2b)), financial security-successfulness (H3a) & H3b)), confidence-assertiveness (H4a) & H4b)), and education-intelligence (H5a) & H5b)).
Robustness analyses 1: random intercept for language instead of countries; robustness analyses 2: excluding women with missing answers to the seriousness question ($n_{excluded}$ = 369); robustness analyses 3: random intercept for language instead of countries and excluding women with missing answers to the seriousness question ($n_{excluded}$ = 369). Graphs display standardized linear age effects, error bars represent 99.5% confidence intervals. Dotted lines indicate the inference criteria for substantial effects *(p* < .005 and |*β*| > 0.10 with *CI*s excluding ±0.10*).*

Table S8

Main results for age effects on parenting intention in the sample of bisexual women

Hypothesis	Outcome	*n*	Countries	Predictor	Age Effect *b [CI]*	Age Effect *β [CI]*	*p*
H6a)	Importance of shared preference for number of children	3,009	92	linear	0.04 [0.02, 0.06]	0.09 [0.04, 0.14]	< .001
H6b)	Preferred level of partner's intention to become a parent	2,493	85	linear	-0.03 [-0.06, -0.01]	-0.08 [-0.14, -0.03]	< .001
H7a)	Importance of shared preference for number of children	3,009	92	linear	0.12 [-0.02, 0.26]	0.27 [-0.05, 0.58]	.02
				quadratic	-0.00 [-0.00, 0.00]	-0.18 [-0.40, 0.14]	.11
H7b)	Preferred level of partner's intention to become a parent	2,493	85	linear	0.00 [-0.15, 0.15]	0.00 [-0.37, 0.37]	.99
				quadratic	-0.00 [-0.00, 0.00]	-0.08 [-0.46, 0.29]	.52

Note. Models for hypotheses H6a and H6b included age as a linear predictor and a random intercept for country. Models for hypotheses H7a and H7b included age as a linear and as a quadratic predictor and a random intercept for country. Sample size and number of countries differ for reasons explained in detail in the section "Sample Characteristics" of the main manuscript. For all hypotheses except for H7a and H7b the raw and the standardized beta coefficient for the linear effect of age are displayed. For H7a and H7b the raw and the standardized beta coefficient for the quadratic effect of age are displayed. In the models including only the linear predictor, the effect of age never reached substantiality ($p < .005$ and $|\beta| > 0.10$ with *CI*s excluding ±0.10).

b = raw beta coefficient, *β* = standardized beta coefficient, *CI* = 99.5% confidence interval.

Fig. S11

Effect size estimates of linear and quadratic age effects on preference for parenting intention for main analyses and three robustness analyses in the sample of bisexual women

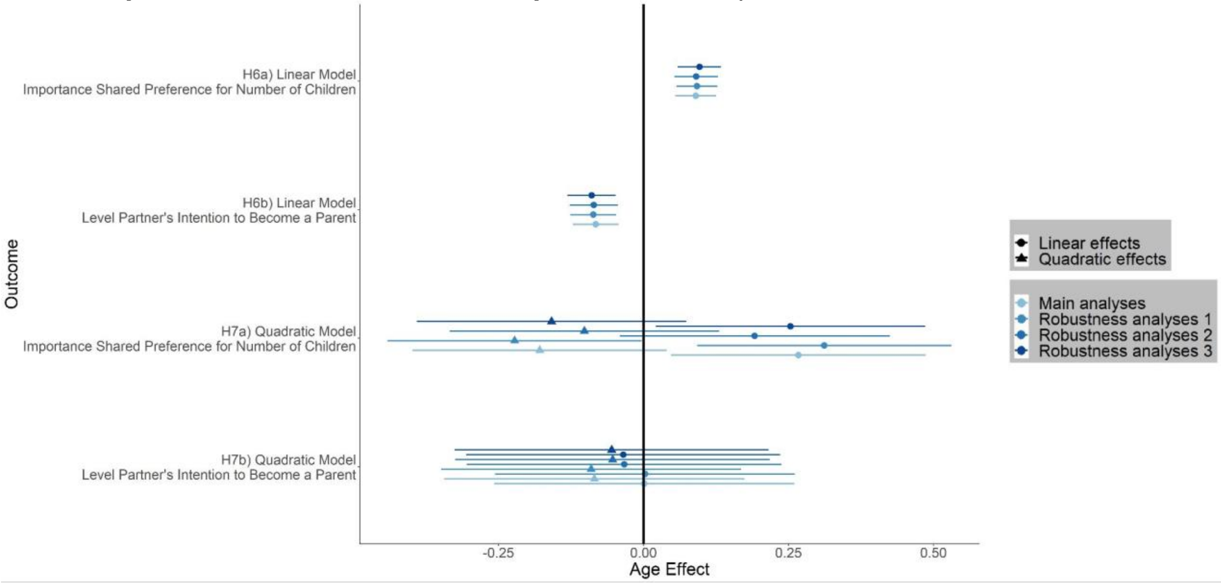

Note. Robustness analyses 1: random intercept for language instead of countries; robustness analyses 2: excluding women with missing answers to the seriousness question ($n_{excluded}$ = 369); robustness analyses 3: random intercept for language instead of countries and excluding women with missing answers to the seriousness question ($n_{excluded}$ = 369). Graphs display standardized linear and quadratic age effects, error bars represent 99.5% confidence intervals. Confidence intervals for effects estimates based on linear models are narrower compared to confidence intervals for effect estimates based on quadratic models because including a quadratic term decreases accuracy of estimates. In the models including only the linear predictor, the effect of age never reached substantiality ($p < .005$ and $|\beta| > 0.10$ with *CI*s excluding ±0.10).

Fig. S12

Preference for parenting intention by a linear and quadratic effect of age controlled for a random intercept for country in the sample of bisexual women

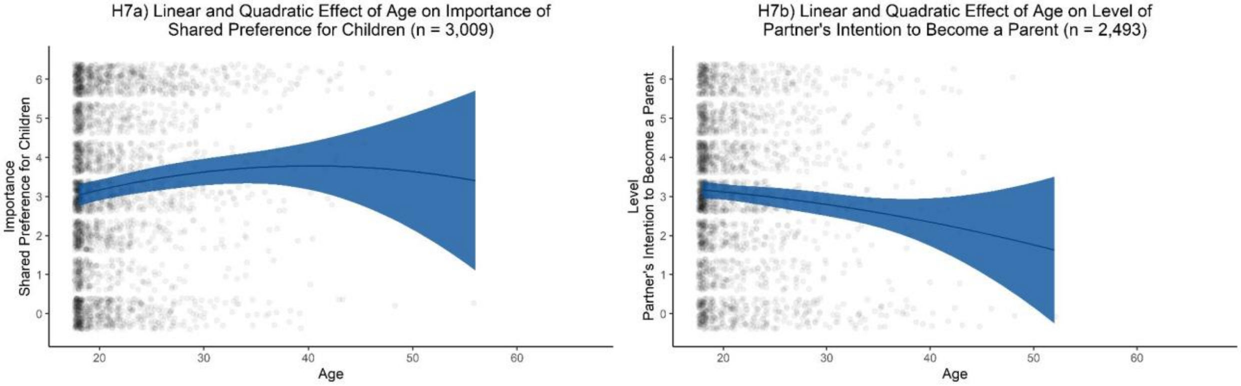

Note. Parenting intention includes importance rating for a partner sharing the preference for number of children (H7a) and preference for the level of partner's intention to become a parent (H7b). Blue areas represent 99.5% confidence intervals.

Table S9

Main results for age effects on ideal age-range in the sample of bisexual women

Hypothesis	Outcome	*n*	Countries	Linear Age Effect *b [CI]*	Linear Age Effect *β [CI]*	*p*
H8)	Age-range deemed acceptable	2,239	84	**0.42 [0.35, 0.50]**	**0.32 [0.26, 0.37]**	< .001
H9)	Youngest age deemed acceptable	2,239	84	**0.34 [0.30, 0.38]**	**0.43 [0.37, 0.48]**	< .001
H10)	Oldest age deemed acceptable	2,239	84	0.08 [-0.00, 0.17]	0.06 [-0.00, 0.12]	.007

Note. All analyses included a linear effect of age as a predictor and a random intercept for country. Sample size and number of countries differ for reasons explained in detail in the section “Sample Characteristics” of the main manuscript. For all hypotheses the raw and the standardized beta coefficient for the linear effect of age are displayed. Bold effect size estimates indicate substantial effects (p < .005 and $|\beta|$ > 0.10 with *CI*s excluding ±0.10).
b = raw beta coefficient, β = standardized beta coefficient, *CI* = 99.5% confidence interval.

Fig. S13

Effect size estimates of linear effects on age-range deemed acceptable and on youngest and oldest age deemed acceptable for main analyses and three robustness analyses in the sample of bisexual women

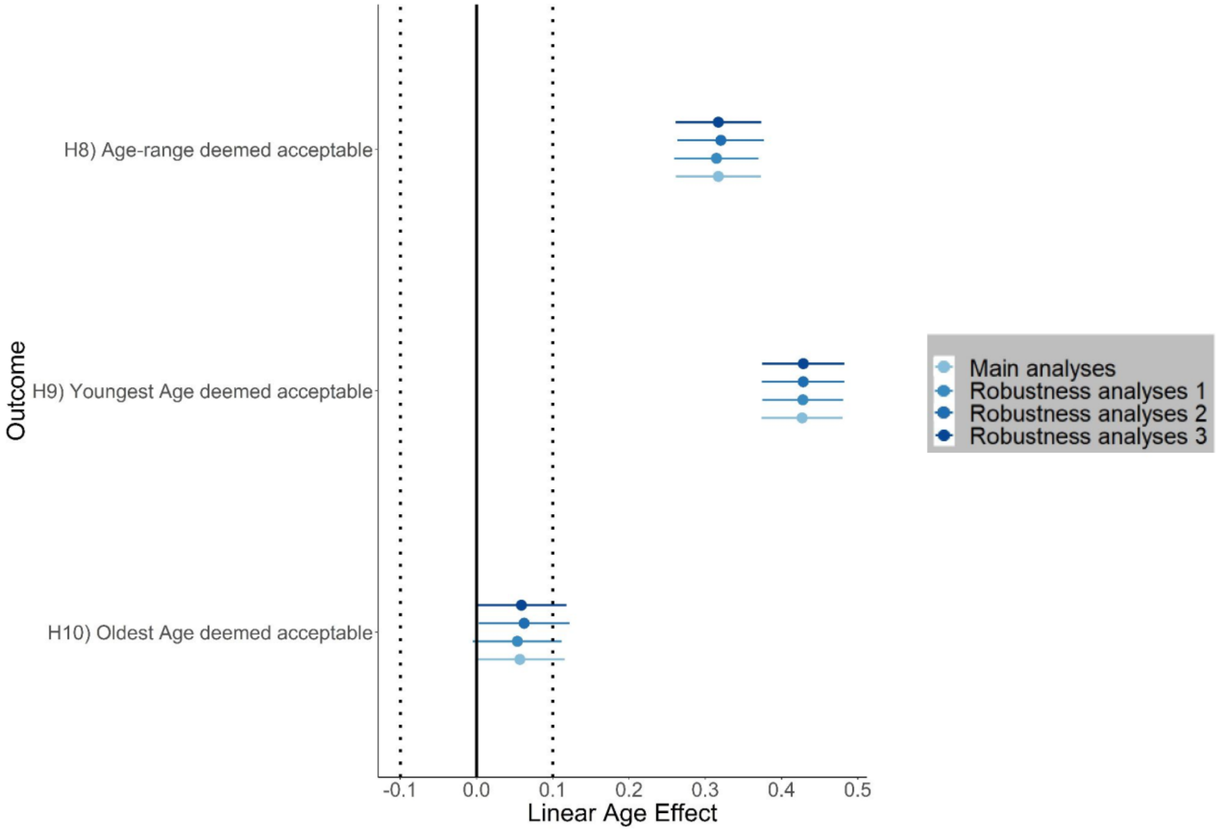

Note. Robustness analyses 1: random intercept for language instead of countries; robustness analyses 2: excluding women with missing answers to the seriousness question ($n_{excluded}$ = 369); robustness analyses 3: random intercept for language instead of countries and excluding women with missing answers to the seriousness question ($n_{excluded}$ = 369). Graphs display standardized linear age effects, error bars represent 99.5% confidence intervals. Dotted lines indicate the inference criteria for substantial effects *(p* < .005 and |*β*| > 0.10 with *CI*s excluding ±0.10*).*

Fig. S14

Age-range deemed acceptable and youngest and oldest age deemed acceptable by own age in the sample of bisexual women

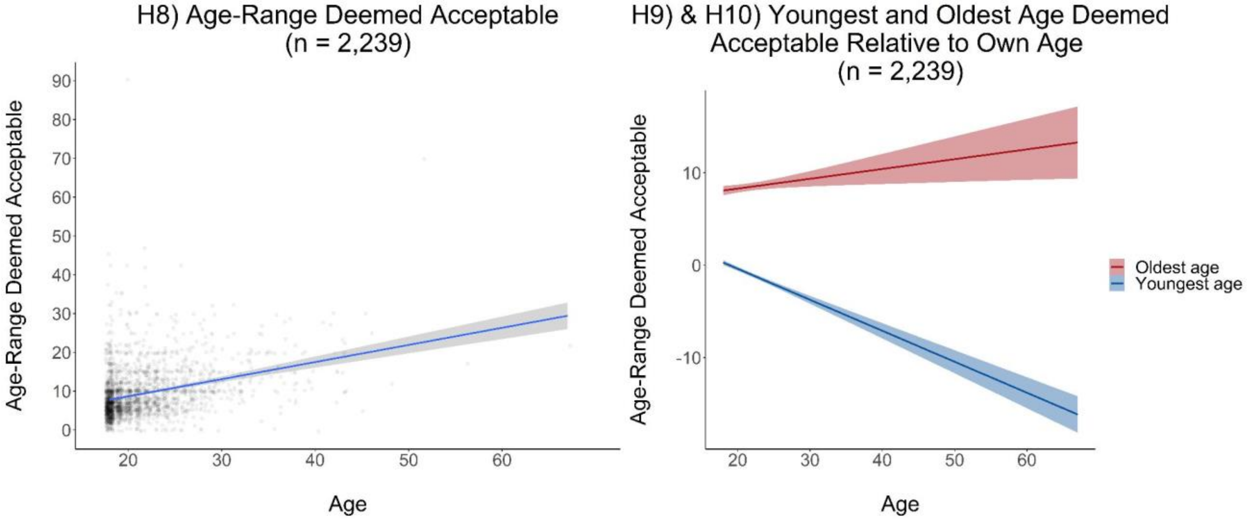

Note. Graphs display means and 99.5% confidence intervals.

References

Simonsohn, U. (2018). Two lines: A valid alternative to the invalid testing of U-shaped relationships with quadratic regressions. *Advances in Methods and Practices in Psychological Science*, *1*(4), 538–555. https://doi.org/10.1177/2515245918805755

Van't Veer, A. E., Vazire, S., Campbell, L., Feldman, G., Etz, A., & Lindsay, D. S. (2019). Preregistration planning and deviation documentation (PPDD). https://osf.io/ywrqe/.
